# Unveiling the therapeutic potential of cabozantinib-loaded poly D,L-lactic-co-glycolic acid and polysarcosine nanoparticles in inducing apoptosis and cytotoxicity in human HepG2 hepatocellular carcinoma cell lines and *in vivo* anti-tumor activity in SCID female mice

**DOI:** 10.3389/fonc.2023.1125857

**Published:** 2023-02-15

**Authors:** Sankha Bhattacharya, Vipan Kumar Parihar, Bhupendra G. Prajapati

**Affiliations:** ^1^ Department of Pharmaceutics, School of Pharmacy & Technology Management, SVKM’S NMIMS Deemed-to-be University, Shirpur, Maharashtra, India; ^2^ Department of Regulatory Toxicology, National Institute of Pharmaceutical Education and Research (NIPER), Industrial Area, Hajipur, Bihar, India; ^3^ Department of Pharmaceutical Technology, Shree S.K. Patel College of Pharmaceutical Education & Research Ganpat University, Mehsana, Gujarat, India

**Keywords:** cabozantinib, polysarcosine, HepG2, flow cytometry, annexin V staining, apoptosis analysis

## Abstract

**Introduction:**

The study aimed to develop a nano-based drug delivery system for the treatment of hepatocellular carcinoma (HCC), a type of liver cancer that accounts for 90% of all liver malignancies. The study focused on the use of cabozantinib (CNB), a potent multikinase inhibitor that targets the VEGF receptor 2, as the chemotherapeutic drug. We developed CNB-loaded nanoparticles made from Poly D, L-lactic-co-glycolic acid, and Polysarcosine (CNB-PLGA-PSar-NPs) for use in human HepG2 cell lines.

**Methods:**

By O/W solvent evaporation method, the polymeric nanoparticles were prepared. The various techniques, such as photon correlation spectroscopy, scanning electron microscopy, and transmission electron microscopy were used, to determine the formulation's particle size, zeta potential, and morphology. SYBR Green/ROX qPCR Master Mix and RT-PCR equipment used to measure liver cancer cell line and tissue mRNA expression and MTT assay to test HepG2 cell cytotoxicity. Cell cycle arrest analysis, annexin V assay, and ZE5 Cell Analyzer apoptosis assay were also performed.

**Results:**

The results of the study showed that the particle diameters were 192.0 ± 3.67 nm with 0.128 PDI and -24.18 ± 3.34 mV zeta potential. The antiproliferative and proapoptotic effects of CNB-PLGA-PSar-NPs were evaluated using MTT and flow cytometry (FCM). The IC50 value of CNB-PLGA-PSar-NPs was 45.67 µg/mL, 34.73 µg/mL, and 21.56 µg/mL for 24, 48, and 72 h, respectively. The study also found that 11.20% and 36.77% of CNB-PLGA-PSar-NPs-treated cells were apoptotic at 60 µg/mL and 80 µg/mL, respectively, suggesting that the nanoparticles were effective in inducing apoptosis in the cancer cells. It can also conclude that, CNB-PLGA-PSar-NPs inhibit human HepG2 hepatocellular carcinoma cells and kill them by upregulating the tumour suppressor genes MT1F, MT1X, and downregulating MTTP, APOA4. Further in vivo antitumor activity was well reported in SCID female mice.

**Discussion:**

Overall, this study suggests that the CNB-PLGA-PSar-NPs are a promising drug delivery system for the treatment of HCC, and further research is needed to investigate their potential in clinical treatment.

## Graphical Abstract

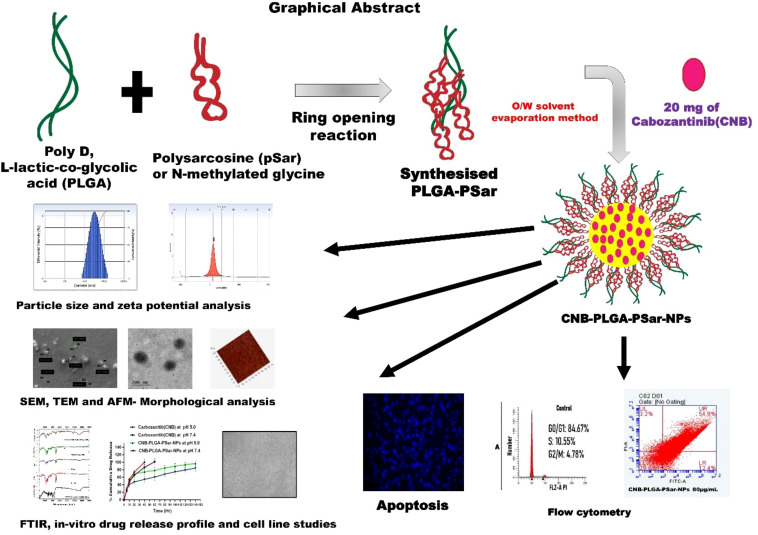

## 1 Introduction

It was often seen that when the same kind of chemotherapeutic drugs (like cytarabine, imatinib, Taxol derivatives, etc.) was used over and over again, their effectiveness was limited, and they caused some unwanted side effects in cancer patients (1–3). To solve this problem, individuals need to work with a different set of molecules. On January 14, 2019, the Food and Drug Administration (FDA) of the United States gave its approval for Cabozantinib to treat hepatocellular carcinoma (HCC) (4, 5). Cabozantinib is a tyrosine kinase inhibitor (RTK); which is often seen overexpressed in the outer layer of the cancerous tissues. From recent studies, it was confirmed that Cabozantinib specifically targets endothelial growth factor receptor types 1 (VEGFR-1) (6), endothelial growth factor receptor types 2 (VEGFR-2) (7), FMS-like tyrosine kinase 3 (FLT-3) (8); which ultimately results in the demotion of angiogenesis and proliferation of malignant tumour (9, 10). As far as liver cancer is concerned (11), it is the sixth most frequently recognized cancer and the fourth leading cause of cancer mortality across the globe (12, 13). As per the WHO report, nearly 782,000 deaths have been recorded due to liver cancer, and surprisingly, liver cancer is more common in men than in women. The most commonly available treatment for liver cancer is surgery and chemotherapy (14). But due to a lack of higher toxicity and lower efficacy, the specific chemotherapeutic drug cannot be administered as such. Therefore, a strong and controlled drug delivery is required to reduce all the associated problems related to chemotherapeutic medicaments (15, 16). The possible ameliorate approach could be the drug-encapsulated polymeric nanoparticular approach (17); which could improve the effectiveness of encapsulation, the drug’s sustainability, and enhance intercellular penetration.

In this study, a new type of non-ionic hydrophilic polypeptide called poly (n-methylated glycine) (18, 19) or Polysarcosine (PSar) was used in the delivery of chemotherapeutic drugs by polymeric nanoparticles; which is the best alternative to corona-forming polymer, poly (ethylene glycol) (PEG) (20). Apart from PSar, the Poly D, L-lactic-co-glycolic acid (PLGA) (21) was also used; due to its excellent biodegradability and biocompatibility properties it is an excellent choice for drug delivery. Therefore, an attempt was made to combine PLGA-PSar nanoparticles encapsulated with Cabozantinib (22, 23) (CNB) with a particle size of<200nm; which ultimately helps in to overcome reticuloendothelial opsonization. In my previous study, “Authors (2021)”; it was observed that Docetaxel loaded Psar-PLGA-NPs could be effective against various human cancer cell lines (U-87 MG, HeLa. C2BBe1, HCT-116, NCI–N87, NCI–H929-Luc-mCh-Puro) and subsequently enhances the blood circulation time (24, 25). As per Blerina Shkodra et al. (26) studies, PLGA-DY-635 (BIM-I) nanoparticles (20-70nm) were prepared; which was comprising with Protein Kinase C Inhibitor; Bisindolylmaleimide (26). The prepared nanoparticles have an effective role in selective liver cancer due to their higher cellular uptake and excellent internalization. Surprisingly, no research has been done on the development of polymer PLGA-PSar-NPs even though the antitumor activities of CNB are increasingly being observed through pharmacological means. In this article, we postulate by findings that modified CNB polymeric nanoparticles might be used as a novel drug delivery system for cancer therapy. Another goal of this research is to use a flow cytometry assay to measure the apoptosis of the HepG2 cell line after it has been treated with nanoparticles. Further, reverse transcriptase polymerase chain reaction (RT-PCR) was done to check for certain gene changes and find a small number of cancerous cells.Nevertheless, lactate dehydrogenase assay, pharmacokinetic studies, tissue distribution studies, *in vivo* cancer activity in SCID female mice, and immunohistochemistry were performed to confirm the biocompatibility, biodistribution, and anti-tumor activity of the prepared CNB-PLGA-PSar-NPs nanoparticles. Therefore, CNB-PLGA-PSar-NPs nanoparticles were formed and tested for the first time in this study against the human HepG2 liver cancer cell line and SCID female mice.

## 2 Materials and methods

### 2.1 Materials

Cabozantinib was a gift sample obtained from Neon Laboratories Ltd, Mumbai, India. Poly D, L-lactide, glycolide, polysarcosine, 2-ethyl hexanoate, methylene chloride, and diethyl ether was purchased from Sigma-Aldrich, Bengaluru, India. Dichloromethane, methanol, polyvinyl alcohol, and d-trehalose was purchased from Sd Fine-Chem Limited, Mumbai, India. Spectra Por S/P 2 Dialysis Membrane was a gift sample from Cole-Parmer^®^, Mumbai, India. From National Centre For Cell Science, Pune, India hepatocellular carcinoma cell line (HepG2) was obtained. Further, Dulbecco’s Modified Eagle Medium (DMEM), Hepatocyte Culture Medium (HHCM), Fetal bovine serum (FBS), tetrazolium salt and propidium iodide (PI), tetrazolium salt (3-(4,5-dimethylthiazol-2-yl)-2,5-diphenyltetrazolium bromide (MTT), 4’, 6 diamidino-2-phenylindole (DAPI), Dimethyl sulfoxide (DMSO), was purchased from HiMedia Laboratories Pvt Ltd, Mumbai, India.

### 2.2 Synthesis of PLGA-PSar

While performing the synthesis of Poly D, L-lactic-co-glycolic acid (PLGA), and Polysarcosine (PSar), the ring-opening reaction was considered (27). At 10 mmHg vacuum pressure and at 160°C, the PSar was dried in at stainless steel container. To enhance the synthesis process, 1.34 g of poly D, L-lactide, and 0.110 g of glycolide were added to PSar (1.25g). In vacuumed condition, the drying process was extended up to 45min, maintaining 160°C. To enhance the reaction, 0.10 g of 2-ethyl hexanoate was added as a catalyst, and further, the reaction was extended up to 6 hours. The resultant copolymer was dissolved in methylene chloride solution, and the solution was ice cold in diethyl ether. The gained product was dried using IKA RV3 V-C Rotary Evaporator; USA.

### 2.3 Preparations of PLGA-PSar-encapsulated cabozantinib nanoparticles

By O/W solvent evaporation method, the polymeric nanoparticles were prepared. In this process, 20 mg of Cabozantinib and 200 mg of PLGA-PSar copolymers were dissolved in a binary mixture of dichloromethane-methanol (8:2). The mixture was further added into ice-cool water containing 1% polyvinyl alcohol (PVA), the resultant mixture was probe sonicated using AI001-Probe Sonicator 650W (Quick lab, India) for 60 seconds for eight times at 50% amplitude, which ultimately results in the formation of O/W emulsion. To remove unentrapped drug, the colloidal nanoemulsion was further centrifuged at 12,000 rpm for 20 min and washed three times with double distilled water. After lyophilization (28) of the resultant dried nanoparticles using 2.5%w/w D-Trehalose at -85°C, the polymeric nanoparticles (CNB-PLGA-PSar-NPs) were stored at -20°C for further use.

### 2.4 Size distribution and zeta potential study

The prepared nanoparticles (CNB-PLGA-PSar-NPs) were measured using DelsaNano C (Beckman Coulter, USA) for particle size, polydispersity index (PDI), and zeta potential (29–31). Photon correlation spectroscopy is the name of the fundamental concept behind this instrument. When an electric field is applied, particles or colloidal solutions emit laser light, which can be measured using this instrument. DelsaNano C can measure particles up to 7 m in diameter and even tiny molecules as small as 267 D in concentrations ranging from 0.001% to 40%. This experiment set the laser weave length to 658 nm at 30 mW. The DelsaNano C can detect zeta potentials between -100mV and +100mV for the sample. The emulsion of the nanoparticles was diluted with 0.50 mL of double distilled water and 0.50 mL of pH 7.4 phosphate buffer solution separately and vortexed for 2 minutes to prevent bubbling. The particle size, zeta potential and PDI were measured at 25°C.

### 2.5 NPs morphology

To determine the morphology of the prepared polymeric nanoparticles, scanning electron microscopy (Hitachi High Technology, SU9000, Japan) was performed (32). With greater stability and instrument resolution at 0.4 nm and 30kV to 1.4 nm and 1kV, 100µL of CNB-PLGA-PSar-NPs were diluted with double-distilled water, and the resulting minuscule quantity was put onto cover slide and dried at room temperature. Using a glass sputter coater, the resultant film was coated with gold under specific vacuumed conditions and scanned under SEM (Carl Zeiss Evo 10 Scanning Electron Microscope). To get proper magnificent images, transmission electron microscopy (TEM) (33) was performed under TEM (Hitachi 7500, Japan). Before performing TEM, the nanoparticles were coated with carbon and placed in Phosphotungstic acid (1%) stained copper grid. The software-generated digital micrograph interprets the evolved images from TEM studies. Atomic force microscopes (AFM) help us learn more about the shape of the CNB-PLGA-PSar-surface (34). The AFM study (AFM5300E, Hitachi) was performed in semiconductor mode and elevated vacuumed condition. During the AFM operation, the AFM prob mobilized in width and lengthwise. The AFM can detect samples within the range of -120°C to 800°C. While operating AFM studies, CNB-PLGA-PSar-NPs suspension was placed in a microscopic slide until the film formed. The kurtosis, average roughness, and average height were calculated for the nanoparticles, and 3D & 2D images were generated for further analysis.

### 2.6 Physical characterization

To determine specific functional groups in Cabozantinib (CAB), free copolymer (PLGA-PSar), and CNB-PLGA-PSar-NPs, the FTIR spectroscopy (Perkin-Elmer, USA) (35) were performed in the frequency range of 400-4000cm^-1^. In order to detect the physical nature of the drug after encapsulation within nanoparticles, differential scanning calorimetry (DSC) studies were performed using DSC 4000 System, 100-240V/50-60Hz (Perkin-Elmer, USA). The samples were scanned, maintaining the temperature range at 25-400°C at a flow rate of 10°C/min. To determine Cabozantinib (CAB), free copolymer (PLGA-PSar), and CNB-PLGA-PSar-NPs thermal stability, Thermogravimetric analysis (TGA) (TA SDT Q600) (36) was performed. At an inert nitrogen environment at 30-500°C and 15°C/min heating rate, the TGA analysis was performed. In respect of temperature, the percentage weight loss has been calculated. To determine the structure of crystalline materials and chemical composition information, XRD studies were performed using Bruker D8 Advance diffractometer at CuKα monochromatic radiation and X-ray source of 40kv and 40mA. With the increment of 0.02°and 2s at each step, at 2θ, the diffractograms were recorded between 20° to 70°.

### 2.7 Encapsulation efficiency

The nanoparticles suspension was placed in a centrifuge and run for 25 min at 10,000 rpm. The obtained nanoparticles were placed in 5mL ethanol for 3h. From the calibration curve, the amount of encapsulated Cabozantinib (CAB) was determined by LAMBDA XLS+ Spectrometer (PerkinElmer Fremont, CA, USA) at 245nm. Percentage drug loading and percentage encapsulation efficiency (%) were calculated from the following equation:


(1) $$\text{Encapsulation efficiency}\left(\%\right)=\frac{\text{The amount of CAB in encapsulated CAB}-\text{PLGA}-\text{PSar}-\text{NPs}}{\text{ Intial amount of CAB used }}\times \;100$$

(2) $$\text{Drug loading}\left(\%\right)=\frac{The\;amount\;of\;total\;loaded\;CAB\;}{\text{The total amount of CAB encapsulated nanoparticles }\left(\text{CAB}-\text{PLGA}-\text{PSar}-\text{NP}s\right)}X\;100$$

After performing three individual experiments, the mean value was calculated (Mean ± SD)

### 2.8 *In vitro* drug release studies and drug deposition studies in dialysis membrane

For *In vitro* drug release studies, Spectra Por S/P 2 Dialysis Membrane (37), 12,000-14,000Dalton 25mm was used. The overnight soaked dialysis membrane in pH 7.4 phosphate buffer solution was tied on dialysis tubes and filled with CNB-PLGA-PSar-NPs suspension comprising 10 mg of Cabozantinib (CAB). After being tied on two sides, the dialysis membrane was placed in 200mL pH 7.4 phosphate buffer solution and kept in sink condition, maintaining the temperature at 37 ± 2°C and 50rpm rotation speed. At specific time intervals, aliquots of the solution have been withdrawn from the donner compartment and replaced with the same amount of fresh pH 7.4 phosphate buffer solution. By compact UV-2600/2700 UV-Visible spectrophotometer, Shimadzu at 245nm, the samples were analyzed. Experiments were performed three times. The same additional process was carried out once more for the evaluation using a pH 5.0 phosphate buffer solution.

Further, various kinetics studies, i.e., zero order, first order, Higuchi, Korsmeyer Peppas model, were performed and concluded that CNB-PLGA-PSar-NPs follow zero-order kinetics. For drug deposition studies in the dialysis membrane, after drug release and diffusion studies, the dialysis membrane was carefully removed and carefully swabbed using pH 7.4 phosphate buffer; three times, the procedure was repeated to ensure no trace of residual particles left onto the diffusion membrane. Further, the dialysis membrane was chopped and entreated in methanol for 48 hr, and the resultant mixture was centrifuged at 50,0000 rpm using Sorvall WX 80 Ultracentrifuge (Thermo Fisher Scientific). The obtained supernatant was analyzed using a compact UV-2600/2700 UV-Visible spectrophotometer, Shimadzu, at 245nm. A standard calibration curve estimated the amount of deposited drug within the dialysis membrane. The purpose of this study was to mimic human epithelial tissue layers.

### 2.9 *In vitro* cytotoxicity assays

For performing *in vitro* cytotoxicity studies, a hepatocellular carcinoma cell line (HepG2) (38, 39) was used. In the presence of a 5% CO_2_ atmosphere, 100 mg/mL streptomycin, and 100 units/mL penicillin, the cells were cultured in a DMEM medium in the presence of a 10% phosphate buffer solution (40). The resultant cells were seeded in 96-well plates in the presence of 100 μL culture medium and kept for 24 hr. Further, the cells were treated with native Cabozantinib (CAB) and CNB-PLGA-PSar-NPs for 24, 48, and 72hr. The culture media was further replaced with Human Hepatocyte Culture Medium (HHCM) (100 μL), which was included with 2% Fetal bovine serum (FBS) and 0.5 mg/mL of yellow tetrazolium salt (3-(4,5-dimethylthiazol-2-yl)-2,5-diphenyltetrazolium bromide, or MTT reagent (41). The temperature was maintained around 37°C during 4hr of incubation. After incubation, the resultant supernatant was replaced with Dimethyl sulfoxide (DMSO). By using a Bio-Rad model 680 microplate reader, absorbance was recorded at 570nm after 30min. The assay was performed in triplicate form.

### 2.10 Apoptotic nuclei measurement using DAPI staining

To investigate internalization and to check changes of nuclei apoptosis, DAPI staining and fluorescence microscopy was performed (42). The selective cell line (HepG2) (1.1 × 10^4^ cells/well) were cultured in 6 well plates and the fixing process extended up to 24hrs.Further HepG2 cells were treated with Cabozantinib (CAB) and CNB-PLGA-PSar-NPs for 24h. Using formaldehyde (4%) and 2% phosphate buffer solution, the cells were washed and fixed. By utilizing Triton-X (0.2%) and 4′,6-diamidino-2-phenylindole (DAPI) (3mg/mL in 2% phosphate buffer solution), the permeabilization and straining of cells were achieved.

### 2.11 Cell cycle arrest analysis

For cell cycle analysis, propidium iodide (PI) (43) staining was used to analyze DNA content in a different phase of the cell cycle. In the presence of 10% phosphate buffer solution, penicillin-streptomycin (1%), the HepG2 cells (5 ×10^4^ cells) were seeded in 12 well plates filled with Roswell Park Memorial Institute (RPMI) 1640 medium and 15% Fetal bovine serum (FBS) albumin. After 24hr of incubation, the cells were treated with Cabozantinib (CAB) and CNB-PLGA-PSar-NPs. The addition of trypsin solution and 70% ethanol would help in trypsinization and the fixation of cells. After 72 hrs, the fixed cells were injected with propidium iodide (PI)and ribonuclease A for 45 min. The cells were further analyzed using a ZE5 Cell Analyzer and 5 lasers (BIO-RAD, USA). The cell fractions of different cell phases were analyzed at 6 ×10^4^ events. All the experiments were performed in triplicate.

### 2.12 Annexin V staining apoptosis analysis

To determine apoptotic and necrotic cells, annexin V assay was used (44). At specific IC_50_ value of free Cabozantinib (CAB) and CNB-PLGA-PSar-NPs, the cells were treated (3×10^5^ cells/well) in 6-well plates. After 48hr, the cells were washed and re-suspended in 1000µL of phosphate buffer solution. In this cell-suspended solution, annexin V-FITC (10 μL) and PI staining solution (10 μL) were added at 25°C and in a dark environment, the incubation process was continued for 10 min; the flow cytometric analysis was performed by considering Annexin V-FITC Apoptosis Staining/Detection Kit (ab14085). The data was entreated by BD Accuri C6 Plus Software.

### 2.13 Real-time reverse transcription PCR assay

RT-PCR (real-time-reverse transcriptase polymerase chain reaction) is a laboratory technique used to detect and quantify specific genes or gene expression in a sample (45). Cabozantinib is a small molecule inhibitor of tyrosine kinases, including MET and VEGFR2. It has been shown to have anti-tumor activity in various cancer types, including renal cell carcinoma and liver cancer (46). The mechanism of action in RT-PCR experiments is likely related to its inhibition of these kinases, leading to decreased tumor cell proliferation and angiogenesis. Additionally, cabozantinib has been shown to have a direct effect on the tumor microenvironment through the inhibition of fibroblast activation and collagen production, which can also contribute to its anti-tumor activity. In RT-PCR experiments on HepG2 cells (a human liver cancer cell line), when treated with Cabozantinib, it is likely that researchers would observe decreased expression of genes associated with tumor cell proliferation and angiogenesis, such as VEGFR2 and MET. The inhibition of these kinases is the primary mechanism of action for Cabozantinib in cancer cells.When Cabozantinib is encapsulated in poly lactic co glycolic acid (PLGA) and polysarcosin nanoparticles, the drug is delivered in a targeted manner to the cancer cells, resulting in increased efficacy and reduced toxicity (47). The exact mechanism of cabozantinib in respect of RT-PCR is not well understood, but it is likely that the drug works by inhibiting the activity of the kinases that are involved in the regulation of gene expression and cell proliferation. Researchers may observe an improved therapeutic effect of Cabozantinib on HepG2 cells when delivered *via* PLGA and polysarcosin nanoparticles, as compared to non-encapsulated Cabozantinib. The HepG2 cells were seeded at 5×10^5^cells/well and further incubated for 24hr at 37°C and in the presence of 5% CO_2_. In the next step, Cabozantinib (CAB) and CNB-PLGA-PSar-NPs in varying concentrations were introduced in the media. After 24hr of incubation, the enthusiast cells were collected, and RNA was separated by Trizol reagent. The RNA was extracted using NanoDrop (45) spectrophotometer at 280nm. For the extraction of RNA, agarose gel (2%) in TBE buffer (1X) was required. A nucleic acid stain called SYBR Safe (Wilmington, De, USA) was utilized for proper visualization of the cells. The SuperScript First-Strand Synthesis System was utilized to synthesize the process of cDNA. The process has been investigated by Real-Time PCR (qPCR) kit (Thermo Fisher Scientific, USA) with 1µg RNA. By instrumenting SYBR Green/ROX qPCR Master Mix (48, 49), the mRNA expression was evaluated. The row data were normalized according to 2^-delta Ct^ in the presence of β-actin; a housekeeping gene.

### 2.14 Evaluation of erythrocyte membrane integrity by lactate dehydrogenase

Erythrocytes produce lactate dehydrogenase (LDH), which may be measured photometrically using the LDH test kit/Lactate Dehydrogenase Assay Kit (Colorimetric) (ab102526) (50). For the LDH investigation, blood was obtained from the neighbouring blood bank. The erythrocyte suspension was made according to the methodology described in our earlier research. In 1mL of erythrocyte solution was treated with specific amount of Cabozantinib (CNB), PLGA-PSar-encapsulated Cabozantinib nanoparticles (CNB-PLGA-PSar-NPs), and placebo (PLGA-PSar-NPs) (51). Positive control sample was created by diluting erythrocyte suspension with 1% Triton-X-100, whereas negative control sample was created by diluting erythrocyte suspension with normal saline solution. LDH was generated spontaneously by incubating 150/UL Lactate dehydrogenase (LDH) with erythrocyte suspension at 37°C. During specific time intervals, such as 2h, 4h, and 8h after suspension, 400µL samples were taken and centrifuged for 20 minutes at 1345xg. LDH was detected at 500nm after a ready-to-use LDH solution was added to the supernatant. The total of LDH was calculated using the following formula.


$$\text{Lactate dehydrogenase }(\text{U}/\text{L})\;=\frac{Asample-A\;negative\;control}{A\;standard\;}\times 150$$

Where, A _sample_ stands for Cabozantinib, PLGA-PSar-encapsulated Cabozantinib nanoparticles (CNB-PLGA-PSar-NPs) incubated erythrocytes absorbance, A _negative control_ indicates, erythrocytes absorbance of sample, which was pre-treated with normal saline solution. The A _standard_ indicates the absorbance of erythrocytes sustention, which were pre-treated with 150/UL Lactate dehydrogenase (LDH) enzyme. The experiment was conducted in triplicate, and the results are presented as mean standard deviation (n=3). The RBC survival rate after nanoparticle injection is dependent on the erythrocyte membrane integrity test. Yuanyuan Guo et al. (52) created poly(d,l-lactide-co-glycolide) (PLGA) nanoplatforms for cancer immunotherapy with erythrocyte membrane encapsulation. With these formulations, cellular absorption *in vitro* was reportedly enhanced (52).

### 2.15 *In-Vivo* anti-tumour efficacy study

The SCID female mice of 8–10 weeks old were used in the *in vivo* effectiveness study (53). SCID stands for severe combined immunodeficiency disorder. Mice were housed in individually ventilated cages with pelleted food, a 12-hour/12-hour dark-light cycle, and unrestricted access to water (supplied ad libitum) in a climate-controlled setting with a temperature range of 25°C ± 3°C. The National Institutes of Health’s guidelines for the care and use of animals were followed when conducting all animal experiments, which included those at Animal House of SPTM, NMIMS, Shirpur, Maharashtra, India. These facilities also complied with CPCSEA (Committee for the Purpose of Control and Supervision of Experiments on Animals) regulations. Mice were subcutaneously injected with cultured HePG2 cells (5x10^6^ cells) in 200µL of RPMI 1640 with 33% Corning Matrigel Basement Membrane Matrix (Sigma-Aldrich, India). When xenograft tumour volumes reached 150mm^3^ (group mean), as instructed by the experimental protocols, the following treatments were given to mice in four groups (n=6): saline, Cabozantinib (CNB) (20 mg/kg), CNB-PLGA-PSar-NPs (20 mg/kg) once weekly (QW) through lateral tailvein. A digital electronic calliper was used to measure the tumor’s size twice a week. To assess toxicity, body weight was monitored twice weekly. Tumour volume was calculated as (width^2^ length/^2^).

#### 2.15.1 Scarification and tumour bulb of liver dissection

The SCID female mice were given thiopental anaesthesia prior to being killed by cervical dislocation. Before being implanted in paraffin wax, liver tissues and tumour bulbs from the descending liver were collected and preserved overnight in 10% formalin (CH_2_O) with a pH 7.4 phosphate buffer solution.

All the animal experiments were performed in Deshpande Laboratory, Bhopal, India, after approval by the Committee for the Purpose of Control and Supervision of Experiments on Animals (CPCSEA) (approval number: 14010/c/11/CPCSEA).

### 2.16 Tissue distribution studies

Tissue distribution studies in mice with SCID are required in anti-liver cancer activity to determine the distribution and concentration of a drug within the body after administration (54). This information is important for understanding how the drug is metabolized and cleared by the body, as well as for determining appropriate dosing regimens. Additionally, tissue distribution studies can help to identify any potential toxicities or side effects associated with the drug in specific organs or tissues. In the case of anti-liver cancer activity, knowing the distribution of the drug in the liver would be particularly important to ensure the drug is reaching its target site and effectively inhibiting the growth of cancer cells. The tissue distribution of a bolus dosage of 20 mg/kg i.v. Cabozantinib (CNB), CNB-PLGA-PSar-NPs was documented. The greatest concentration of CNB was found in liver parenchymal hepatocyte tissue. The presence of CNB in the tissues of other essential organs, such as the heart and kidney, suggests that CNB distribution is indirectly dependent on the blood flow and perfusion rate of the organs. The increased affinity of CNB in the liver and pancreas enables the medication molecule to effectively treat liver cancer. However, limited expression of CNB in brain endothelial cells suggests that polymeric nanoparticles are unable to traverse the blood-brain barrier without difficulty.

### 2.17 Pharmacokinetic study

06 Male Albino Wister rats (200 to 250 g) are separated into two groups (n=3) for the purpose of conducting pharmacokinetic studies (55). For up to 72 hours, a comparative plasma concentration-time profile of Cabozantinib (CNB), CNB-PLGA-PSar-NPs was analysed. The first group was administered 20 mg/kg CNB *via* intravenous bolus with 0.9% w/v sodium chloride and 10% Methanol (for improving solubility). The second group received an intravenous bolus dose of 20 mg/kg CNB-PLGA-PSar-NPs with 0.9%w/v sodium chloride and 10% methanol (for improving solubility). Using the tail vein of rats, 500µL of blood samples were collected at 0, 0.5, 1, 2, 4, 8, 16, 24, 48, and 72 hours from three animals in each group. Blood samples were stored in a microcentrifuge tube and centrifuged at 7,500 rpm for 30 minutes at 25°C to separate supernatant plasma. The collected samples were analysed using the RP-HPLC system and stored at -80°C (1290 Infinity II LC System, Agilent, USA). Using kinetica 5.0 pharmacokinetics software, the obtained RP-HPLC variables were interpreted in terms of their pharmacokinetics (Thermo Fisher Scientific, Yokohama)

### 2.18 Immunohistochemistry

Paraffin 10µm thick sections of xenograft HePG2 cancer cell tissues embedded in liver parenchyma blocks. The blocks were imaged in a 10 mM citrate buffer solution and autoclaved for 20 minutes to extract antigens. After heating the section slide blocks, they were allowed to cool for 1.5 hours at room temperature. The sectioned slides were then incubated overnight at 4°C with a primary antibody against Hepatocyte specific antigen (Hep Par 1) (91091816- SK-HEP-1, Sigma-Aldrich, Bengaluru, India). Further, section slides were incubated with enzyme-linked immunosorbent assay (ELISA) horseradish peroxidase (HRP) conjugated Goat anti-Rabbit IgG (H+L) secondary antibody (Thermo Fisher Scientific, Vadodara, Gujarat) for 1hr followed by treatment with 120µL 30-3-diaminobenzide (Dako). For analysing the results of Immunohistochemistry, five square zones (400 m x 400 m) were measured and averaged for the number of stained cells in each sectioned slide. Using GraphPad Prism 9 (San Diego, California) and student t-test statistical analyses were performed.

### 2.19 Statistical evaluation

The statistical calculation and interpretations were performed by GraphPad Prism 9.0.0.121 (GraphPad Software, San Diego, CA 92108). The experiments were performed thrice and defined as the mean ± standard deviation (SD). For comparison between two groups, student t-test and for multiple groups, ANOVA was applied. The p<0.05 was recognized as statistically significant.

## 3 Results and discussion

### 3.1 Preparations of PLGA-PSar-encapsulated cabozantinib nanoparticles

To improvise local therapeutic effects, biodegradable depots in nanosized polymeric particle forms could be the best non-invasive approach for drug delivery applications. Biodegradable synthetic polymers such as, lactic-*co*-glycolic acid (PLGA) & Poly-Sarcosine (PSar) were used in this experiment for synthesizing polymeric nanoparticles. The polymers are made up of single monomers (56); therefore, the excretion from the body would be effective, and hence, they possess low toxicity (57). These nanosized polymeric nanoparticles could play a fundamental role while delivering a drug to the targeted tissues or oranges. According to Mohadese Alirezaei et al. (58) Vulgaris oil-containing PLGA-chitosan-folic acid nanoparticles (AVEO-PCF-NPs) exhibit excellent anticancer properties against the HT-29 cell line (58). Furthermore, Chinese yam polysaccharides PLGA nanoparticles stabilized Pickering emulsion can be prepared by using 5 mg/mL of PLGA. Due to the presence of PLGA, CYP-PPAS emulsion exhibits outstanding stability at 4°C and 37°C for 28 days, according to Yue Zhang et al. (59). It is clear from both research findings that PLGA exhibits excellent stability and can be a suitable polymer for anticancer drug delivery.

The intrinsic characteristics of polymers are investigated during the selection of any biological macromolecules. These biocompatible polymers help to encapsulate biodegradable particles. For targeting tissues and cells, these agents act as a depot. These kinds of delivery possessed higher non-toxic effects, durability, sustainability and controlled release quality in blood. Most importantly, polymeric nanoparticles enhance the hydrophilicity as well as zeta potential. Polymers become water-soluble when they contain polar or charged functional groups (60). Water or other polar substances can interact with polymers or dissolve them. Further, the presence of PVA in the nanoparticles could influence the zeta potential of the nanoparticles (61).

Nevertheless, polymeric nanoparticles improvise bioadhesive properties and release the nature of the formulations. Therefore, in this experiment, biocompatible PLGA-PSar copolymers are used for the encapsulation. The temperature-sensitive PLGA-PSar copolymers remain in a solution state between 0°C to 20°C and shift to a gel state between 37°C to 77°C. The copolymer is comprised of hydrophilic PSar and a hydrophobic moiety; As a result, the copolymers might function as amphiphilic copolymers. The preparation of oil/water emulsion can instantly be formed when the organic phase is incorporated into the aqueous phase in the presence of prob sonication. The polymeric nanoparticle’s reaction rate can be enhanced due to the presence of Gibbs-Marangoni effects (62). The solidification process of nanoparticle preparation helps to build condensed nanoparticles. It was observed during initial analysis to prevent nanoparticle aggregation that emulsifiers within the water in oil interphase restrict nanoparticle aggregation. The amphiphilic copolymer and stabilizer, PVA, has been added as an emulsifier during nanoparticle formulation. The hydrophobic portion (undissociated vinyl acetate portion) of PVA diffused into the organic phase and was eventually trapped inside the nanoparticles. The hydrophilic portion of PVA remains active in the surroundings of hydrophilic segments of nanoparticles, and stability persists due to steric hindrance. PVA contains many hydroxyl groups, forming hydrogen bonds with the water molecules and making it highly soluble in water. The molecular weight and degree of hydrolysis of PVA determine its solubility (63).

### 3.2 Particle size and morphological studies

**Figure 1:**
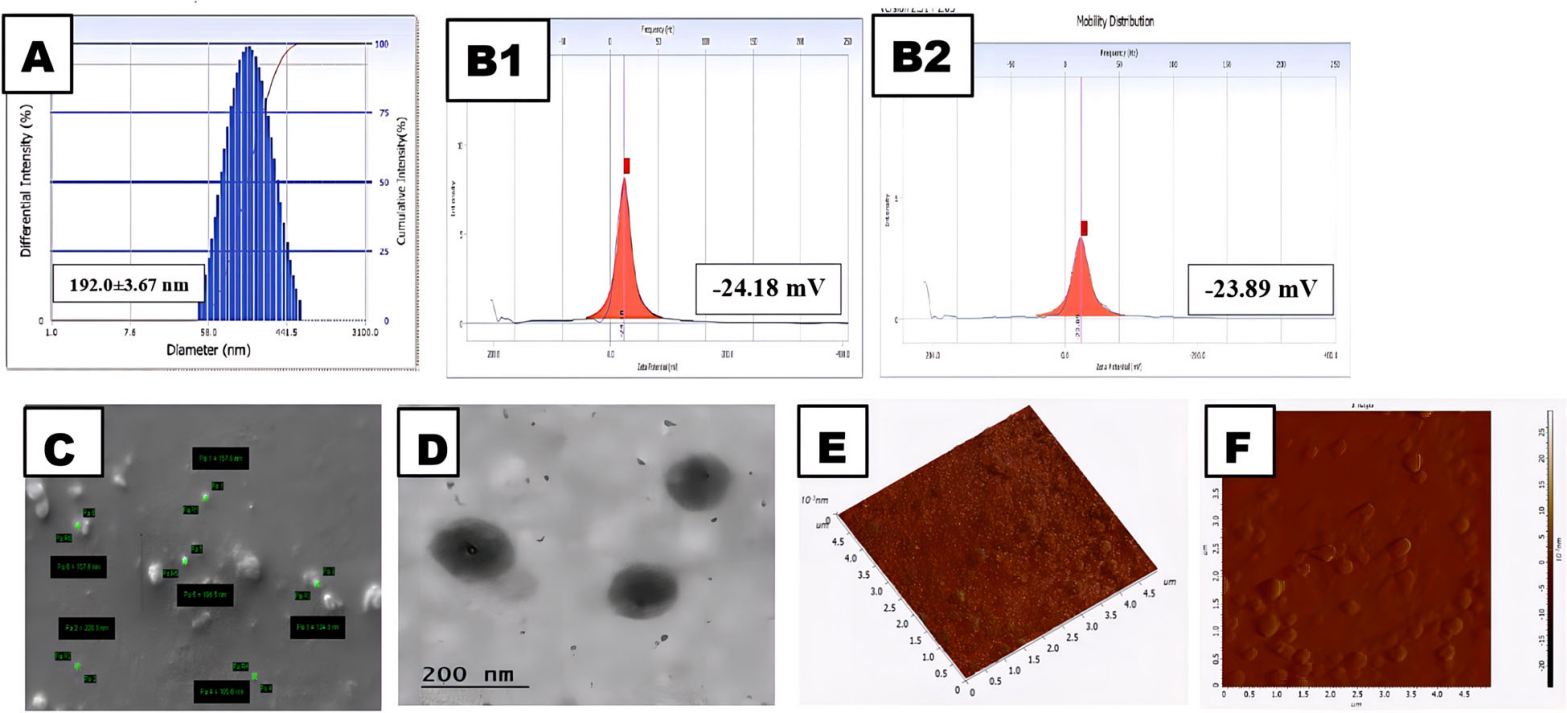
Physical characterization of the CNB-PLGA-PSar-NPs: **(A)** Size of the NPs. **(B1)** Zeta potential of the NPs in water; **(B2)** Zeta potential of the NPs in Phosphate buffer solution (pH 7.4).**(C)** Field emission scanning electron microscopy (FE-SEM) images of the NPs. **(D)** Transmission electron microscopy (TEM) of the NPs **(E)** Atomic force microscopy image of NPs in 3D **(F)** Atomic force microscopy image of NPs in 2D.

The *in-vitro* drug release profile is very significant; the *in-vitro* biodistribution and cellular absorption of the prepared nanoparticles display a characteristic and optimum behavior. The CNB-PLGA-PSar-NPs particle size, PDI, and zeta potential could therefore play a crucial role in the stability and all distinctive & optimum behavior of the nanoparticles. From **Figures 1A, B**, the average particle diameters of CNB-PLGA-PSar-NPs were found to be 192.0 ± 3.67 nm with a PDI of 0.128. The zeta potential was found to be -24.18 ± 3.34 mV in double distilled water; however, in pH 7.4 phosphate buffer solution, the zeta potential of the nanoparticles was found to be -23.89 ± 1.07 mV. From the particle size distribution curve, it can conclude that the exact binomial distribution or Gaussian distribution had been recorded. Rifampicin-loaded PLGA nanoparticles (RIF-NPs) can be formed using the nanoprecipitation method, according to Eva Snejdrova et al. (64), to treat musculoskeletal infections (64). The obtained nanoparticles range in size from 200 to 380 nm and have a negative zeta potential. Paclitaxel and Lapatinib dual-loaded chitosan-coated PLGA nanoparticles with a particle size range of 157.4 ± 9.2 to 263.5 ± 13.5 nm were created, according to Subrahmanyam Pitchika et al. (65). These two studies suggest that PLGA can be used to create nanoparticles with sizes between 180 and 400 nm; the results are consistent with our recent studies. During nanoparticles size measurement, it was observed that after encapsulation of Cabozantinib inside of the polymeric nanoparticles, the core diameter increases for the nanoparticles, and hence Cabozantinib encapsulated nanoparticles are more significant in size than blank nanoparticles. Due to the higher negative zeta potential of the formulation, the formulation could have a longer circulation half-life. Due to the deportation of H+ ions from the carboxylic group of PLGA, the negative zeta potential has emerged. Since zeta podetial is a surface phenomenon, it was observed that zeta potential and particle size are inversely proportional; which means increasing PLGA-PSar concentration on the outer surface of the nanoparticles would lead to a decrease in zeta potential. Hence, higher negative zeta potential could lead to the formation of smaller particle sizes and, thus, higher migration capacity observed in nanoparticles, which ultimately provides good stability in colloidal dispersion. The drug loading capacity of CNB-PLGA-PSar-NPs was found to be 14.67 ± 3.86%, and encapsulation efficacy was found to be 76.35 ± 2.76%. The monodispersed nature of the polymeric nanoparticles was further investigated using a scanning electron microscope (SEM). The SEM results suggest that there is a persistent similarity of particle size results obtained from the particle size analyzer with SEM results. The homogeneity of the nanoparticles with core structure was observed in SEM images with the highest diameter of 228.8 nm. It measured the lowest diameter at 124.3nm (**Figure 1C**). The average particle size obtained by Delsa Nano C instruments (Beckman Coulter, USA) was found relatively more significant than the results obtained from SEM (Carl Zeiss Evo 10 Scanning Electron Microscope) analysis; which is due to the presence of dehydration environment during the time of the SEM experiment. **Figure 1D** represents the TEM analysis of CNB-PLGA-PSar-NPs, which indicates perfect spherical-shaped drug-loaded nanoparticles (<200nm). From scanning force microscopy or atomic force microscopy, the non-destructive surface of nanomaterials, kurtosis (0.712), skewness (0.264), and roughness (4.134), can be measured using NOVA software. From **Figures 1E, F** the average height of the nanoparticles was found to be 198.67 ± 0.32 nm; the obtained particles’ average height had significant similarity with the results obtained from SEM, TEM, and DLS studies, indicating the vital significance of the particles in different measurement conditions. The AFM report suggests the spherical and symmetrical nature of the prepared CNB-PLGA-PSar-NPs surface.

### 3.3 Fourier transform infrared spectroscopy

**Figure 2:**
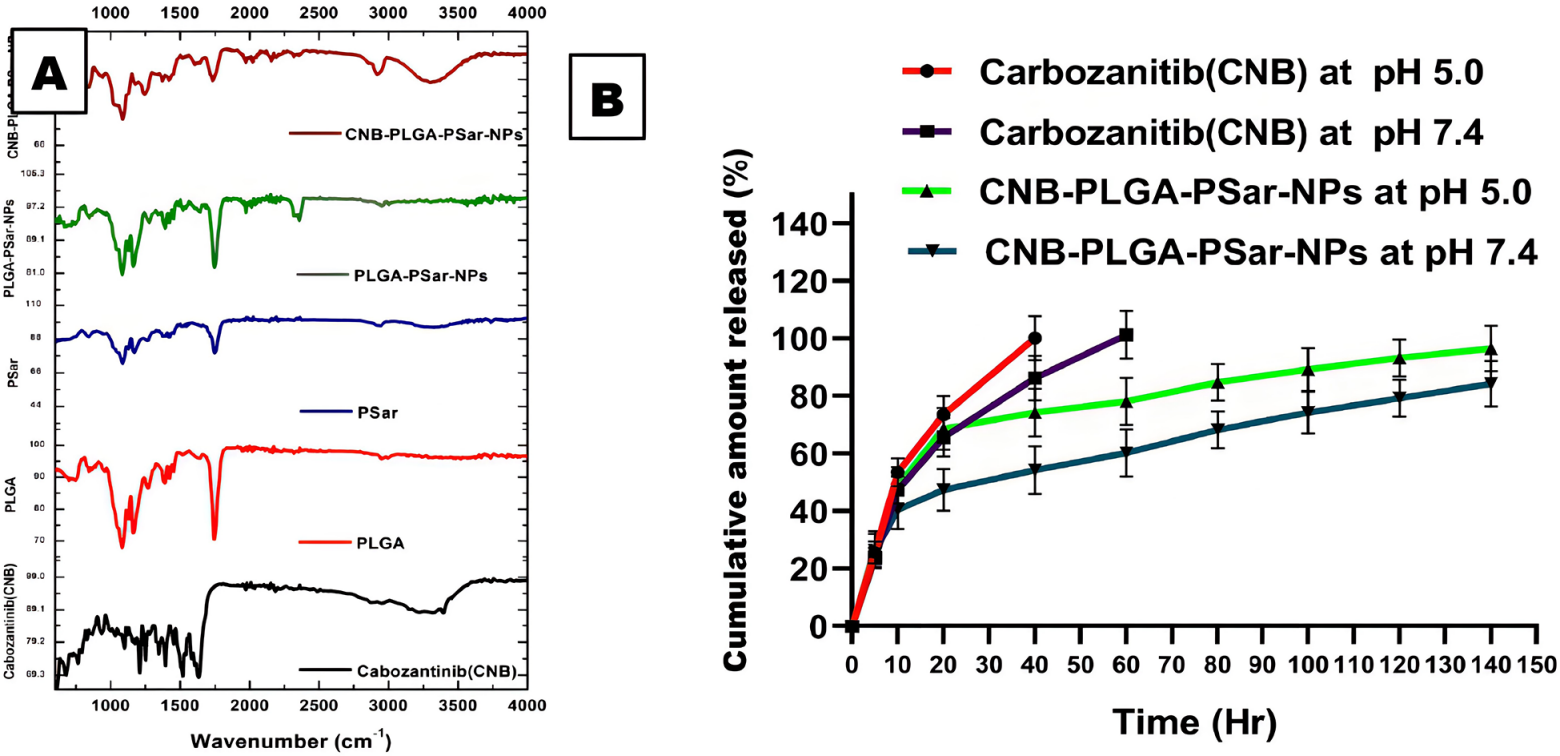
**(A)** FTIR data of Cabozantinib (CNB), PLGA, PSar, PLGA-PSar-NPs, CNB-PLGA-PSar-NPs. **(B)** *In vitro* release kinetics of CNB from CNB-PLGA-PSar-NPs in PBS at pH 5.0 and 7.4 and data are presented as mean ± SD (n= 3).

The main intention of FTIR spectroscopy was to identify the complete loading capacity of the drug into the carrier. The FTIR spectroscopy of Cabozantinib (CNB), PLGA, PSar, PLGA-PSar-NPs (without drug), and CNB-PLGA-PSar-NPs (with drug) were displayed in **Figure 2A**. In Cabozantinib (CNB) spectra, at 1221.904 cm^-1^ N-O aromatic stretch was observed, C=O stretch was observed at 1646.66 cm^-1^, at 3411.42 cm^-1^ aromatic O-H stretch was observed. poly D, L-lactic-co-glycolic acid (PLGA) shows characteristic peak at 1606.61 cm^-1^, 1742.85 cm^-1^ & 2977.14 cm^-1^ indicating the presence of carboxylic acid C=O stretch, ester C=O stretch and carboxylic acid dimer O-H stretch respectively. The polysarcosine (PSar) also shows a characteristic peak at 1723.80 cm^-1^, 3305.71 cm^-1,^ which indicates the presence of aldehyde C=O stretch & O-H stretch. The placebo formulation (PLGA-PSar-NPs) showed strong C=O stretch at 1733.33 cm^-1^; however, no significant characteristic peak was observed between 2750 cm^-1^ to 4000 cm^-1^, indicating the complete encapsulation of PSar and PLGA within the placebo nanoparticles. The final CNB-PLGA-PSar-NPs shows a characteristic peak at 1761.90 cm^-1^, 2939.04 cm^-1^, 3315.23 cm^-1^ indicating the presence of monomeric C=O stretching, carboxylic acid dimer OH stretch, and alkynes C-H stretch. The appearance of CNB-PLGA-PSar-NPs FTIR spectra indicates the interactions of Cabozantinib (CNB), PLGA, and PSar.

### 3.4 *In vitro* release kinetics of cabozantinib

For the Hepatocellular carcinoma (HCC) treatment, it is essential to have prolonged and consistent release of Cabozantinib (CNB) from CNB-PLGA-PSar-NPs. The release of Cabozantinib (CNB) from the polymeric nanoparticles was estimated in pH 5.0 and pH 7.4 at 37°C. It was palpable from the release profile; the drug has shown an initial bust affect up to 4^th^ hour of drug release. This burst release signifies the presence of drug content onto the surface of polymeric nanoparticles, which elevates initial drug release. After 5^th^ hr a steady state concentration was reached at both the pH and hence prolonged release drug profiling was witnessed at 140hr. It was observed that at pH 7.4, the drug release from CNB-PLGA-PSar-NPs was found to be much more profound as compared to drug release at pH 5.0. At 140^th^ hours in pH 7.4 the Cabozantinib shows they release up to 84.25 ± 7.89% whereas else, at pH 5.0, Cabozantinib from the nanoparticles was found to be 96.56 ± 7.89%. It was observed that during the time of cellular internalization at pH 5.0, Cabozantinib (CNB) shows higher releases as compared to pH 7.4; which may be due to the acidic nature and pH-sensitive behavior of the cancerous cell surface (**Figure 2B**). The pinocytosis and endocytosis are responsible for cellular internalization of nanoparticles. The prolongation in drug release in the tumour site could enhance the effectiveness of chemotherapeutic drugs near to the cancerous cells. Further, the nanoparticular approach incepted in this research could enhance the selectivity profiling of the medicaments, and in lower dosage regimes, it can effectively target cancerous cells omitting healthy cells. The prolonged-release profiling of the nanoparticles could enhance the delivery of drug in constant manner into the cancerous cells, which ultimately leads to an increase the cytotoxicity effects.

### 3.5 *In vitro* cytotoxicity

**Figure 3:**
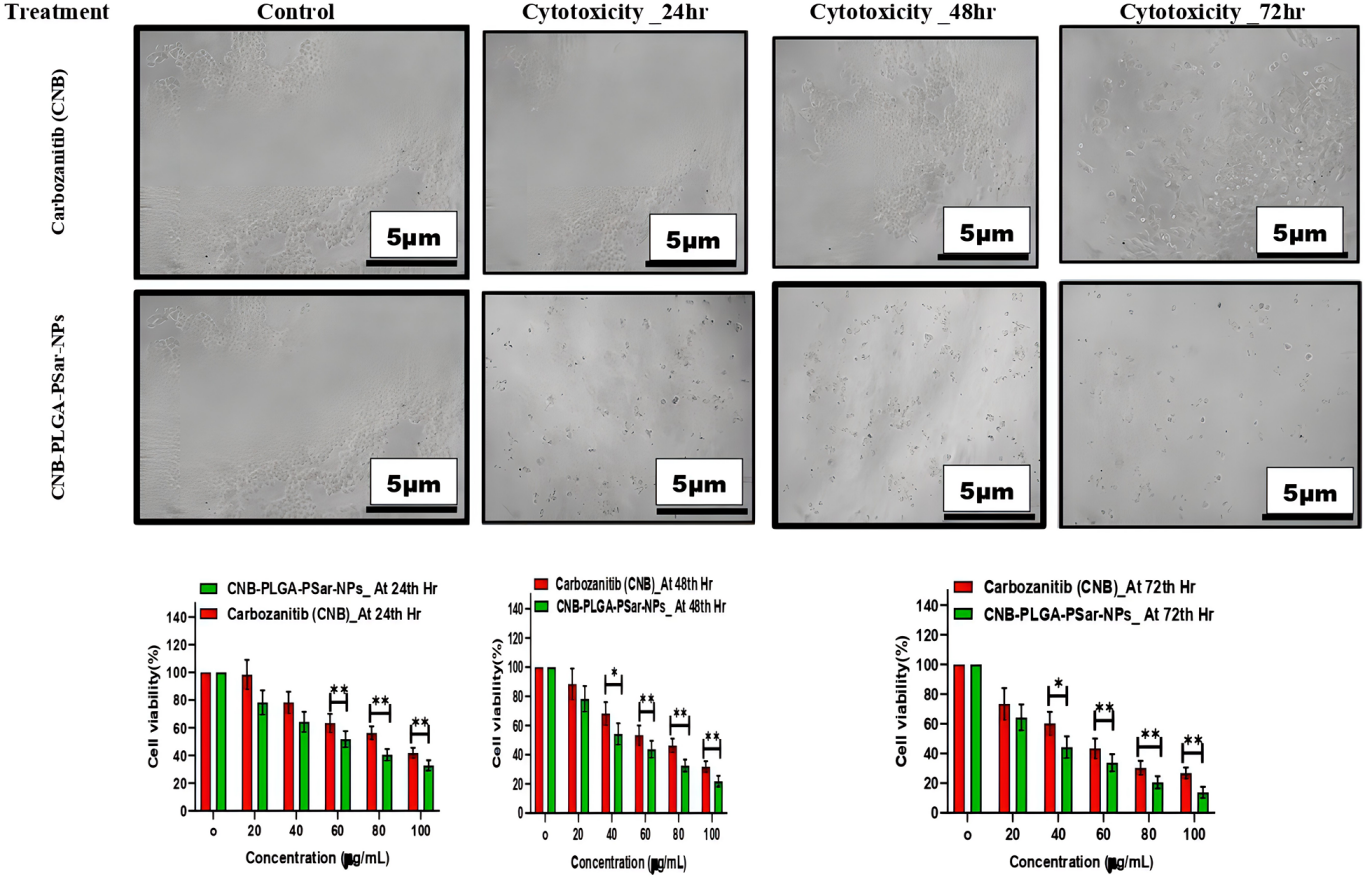
Effect of different concentrations of CNB-loaded CNB-PLGA-PSar-NPs and free CNB on the viability of hepatocellular carcinoma cell line (HepG2) cells after 24, 48, and 72 h incubation. The figure shows that CNB-loaded CNB-PLGA-PSar-NPs are more anti-proliferative when compared with CNB (*p < 0.05). The cell viability was determined by MTT assay. These experiments are performed thrice mean ± SD; n= 3, *p < 0.05, **p < 0.01, where CNB group is considered as control.

The 3-(4,5-dimethylthiazol-2-yl)-2,5-diphenyl tetrazolium bromide (MTT) (66) assay helps to determine the cytotoxicity effects of free Cabozantinib (CNB) and CNB-PLGA-PSar-NPs against hepatocellular carcinoma cell line (HepG2). The IC_50_ value of Cabozantinib (CNB) loaded polymeric nanoparticles (CNB-PLGA-PSar-NPs) was found to be 45.67 μg/mL, 34.73 μg/mL, and 21.56 μg/mL respectively, when exposed at 24hr, 48hr and 72hr. **Figure 3** suggests that in time and dose-dependent manner, the free Cabozantinib (CNB) and Cabozantinib (CNB) loaded polymeric nanoparticles (CNB-PLGA-PSar-NPs) show anticancer effects against hepatocellular carcinoma cell line (HepG2) (67, 68). Due to the control and slow-release properties of the drug from the polymeric nanoparticles, it exhibits a higher cytotoxicity profile as compared to free Cabozantinib (CNB). Therefore, polymeric nanoformulations could be the best formulation approach to deliver anticancer drugs in targeted site. As per Gao Feng Liang et al. (69) research, the suppression effects of the miRNA can be enhanced when PLGA-based gene delivery is performed for HePG2 cells. The PLGA nanoparticles could be the best possible vector carriers for miRNA with significant transfection efficiency (69). These nanoparticles show good serum stability against commercially available liposomes. Cabozantinib is a tyrosine kinase inhibitor that tends to block both the MET and VEGF pathways from functioning, as Qiuhong Yang et al. (70) study confirmed (70). The cabozantinib-loaded micelles outperformed the free cabozantinib solution in terms of intracellular accumulation and cytotoxicity in human glioblastoma cancer cells and non-small lung cancer cells. These findings imply that the micellar form of cabozantinib may be an effective nanocarrier for cancer therapies. As per Xiaoling Nie et al. (71) research by using the nanoprecipitation method, SP94 peptide surface grafted poly (ethylene glycol)-poly (lactic-co-glycolic acid) biodegradable polymeric nanoparticles were prepared for the delivery of cryptotanshinone (71). The SP94 peptide surface grafted polymeric nanoparticles significantly increase cellular apoptosis and cytotoxicity against hepatocellular carcinoma cell line (HepG2). As per Xiaobo Yang et al. (72), Cabozantinib has a significant effect against Hepatocellular Carcinoma (72). However, no studies have been reported so far which emphasized lactic-co-glycolic acid (PLGA) & Poly-Sarcosine (PSar) encapsulated Cabozantinib (CNB) nanoparticles’ *In vitro* cytotoxicity effects against hepatocellular carcinoma cell line (HepG2). The controlled release pattern of CNB-PLGA-PSar-NPs enhances the therapeutics effects due to its specific pathways and maintaining higher intercellular concentration.

### 3.6 DAPI staining

**Figure 4:**
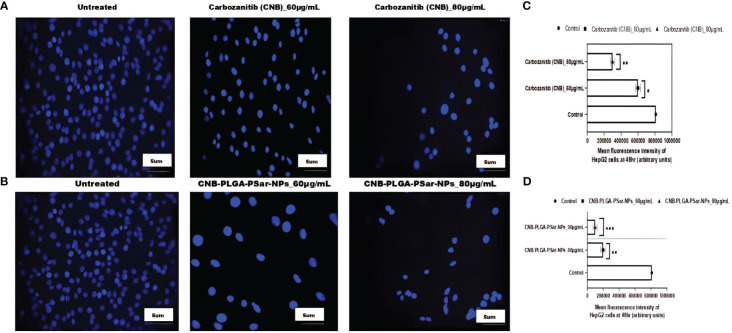
Carbozanitib (CNB)-induced apoptosis and DNA damage in hepatocellular carcinoma cell line (HepG2) cells as examined by DAPI staining. HepG2 cells exposed to different concentrations of CNB-loaded CNB-PLGA-PSar-NPs and free CNB for 48 h and apoptosis was determined by DAPI staining by fluorescence microscopy. **(A)** represented the control (untreated), CNB 60 μg/mL, and CNB 80 μg/mL, respectively. **(B)** represented the control (untreated), CNB-PLGA-PSar-NPs 60 μg/mL, and CNB-PLGA-PSar-NPs 80 μg/mL, respectively. **(C)** Mean fluorescence intensity of HepG2 cells at 48hr when treated with 60 and 80 μg/mL of CNB. **(D)** Mean fluorescence intensity of HepG2 cells at 48hr when treated with 60 and 80 μg/mL of CNB-PLGA-PSar-NPs. Data are presented as mean ± SD. The graph represents data from two independent experiments. p<0.05, student t-test.

To investigate the cell viability of hepatocellular carcinoma cell line (HepG2) accurately after administration of free Cabozantinib (CNB), and Cabozantinib loaded polymeric nanoparticles (CNB-PLGA-PSar-NPs), the treated cells were stained with 4′,6-diamidino-2-phenylindole, (DAPI) reagent and visualized under fluorescence microscopy to check resultant morphology. The section shows circular-shaped cells without any shattering (**Figures 4A, B**). The fluorescence intensity of particle uptake was measured and reported in **Figures 4C, D**. However, small hemispherical nuclei were observed when cells were treated with free Cabozantinib (CNB) and CNB-PLGA-PSar-NPs. The higher dose of free drugs and nanoparticles shows a significant increase of apoptosis. The CNB-PLGA-PSar-NPs treated cells show dramatical apoptosis against HepG2 cells as compared to free Cabozantinib (CNB). Altogether, the viability of the HepG2 cells decreases when treated with higher concentrations of CNB-PLGA-PSar-NPs due to the persistent DNA damage.

### 3.7 Cell cycle arrest analysis

**Figure 5:**
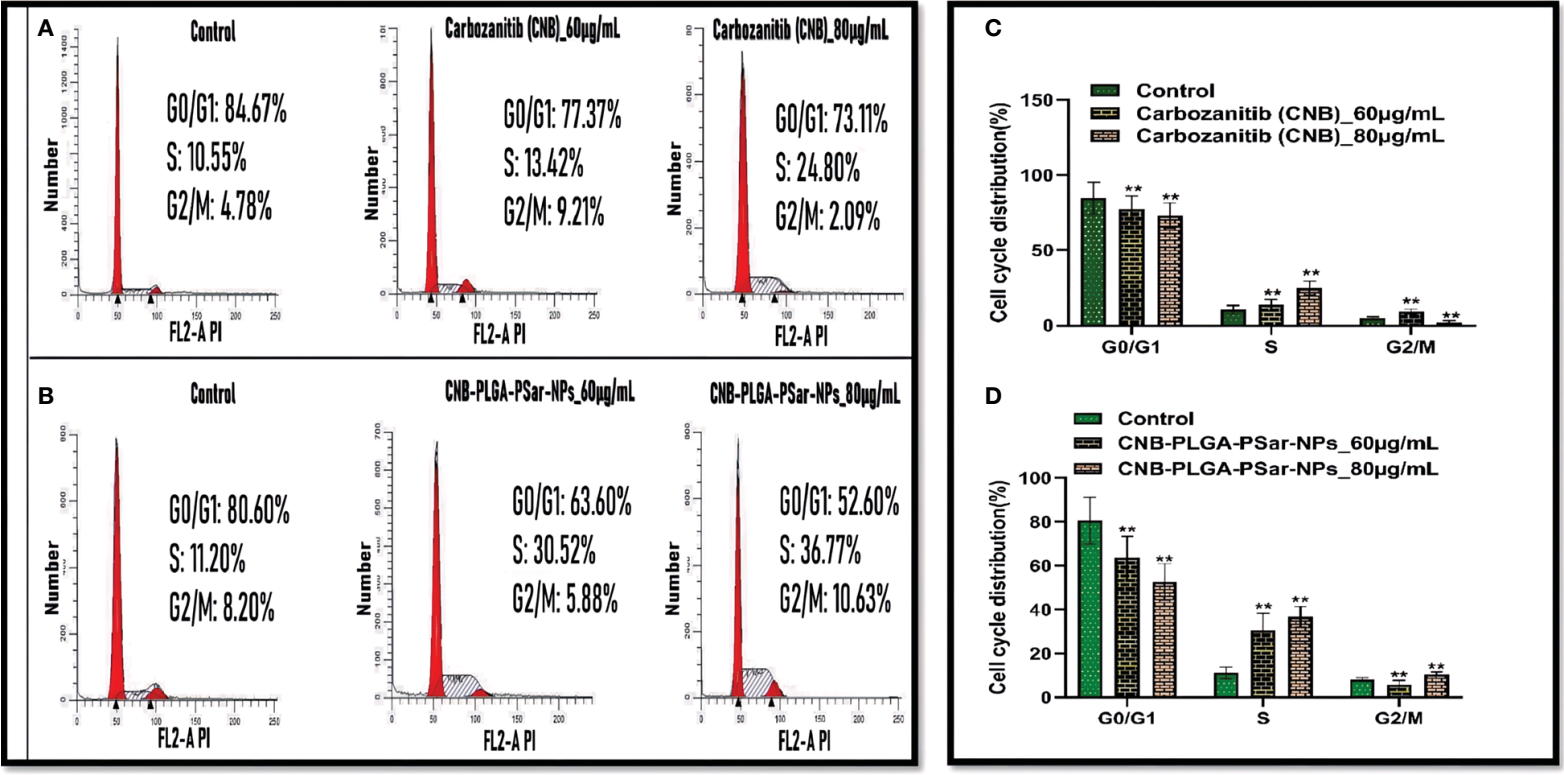
Flow cytometry was used to analyse cell cycle distribution and DNA content in HepG2 cells treated by CNB-loaded CNB-PLGA-PSar-NPs and free CNB for 48 h **(A, B)** The cells were stained with PI and the cell cycle stage was defined by DNA content. **(C, D)** The percentages of cell populations in the G0/G1, S, and G2/M phases was determined by gating during the flow cytometry experiment for control, free CNB and CNB-loaded CNB-PLGA-PSar-NPs at 60 µg/mL & 80 µg/mL concentration. These experiments are performed thrice mean ± SD; n= 3, **p <0.05, where untreated group is considered as control.

It is essential to measure Cabozantinib (CNB) and CNB-PLGA-PSar-NPs mediated growth inhibition and to identify its specific molecular mechanism by cell cycle arrest studies. In this study, the specific DNA content of HepG2 cells was analyzed. The study was designed for 48 hr, where 60 and 80 µg/mL Cabozantinib (CNB) and CNB-PLGA-PSar-NPs were exposed in HepG2 cells. The dose-depending cell cycle arrest can be witnessed for Cabozantinib (CNB) and CNB-PLGA-PSar-NPs (**Figures 5A–D**). The drug and the formulation have DNA synthesis inhibitor properties, as cycle arrest data suggest that the mitotic division was passed by the cells but arrest can be witnessed in S phase. The percentage of the S phase was elevated from 10.55% to 24.80% for Cabozantinib (CNB) and 11.20% 36.77% for CNB-PLGA-PSar-NPs. The greater DNA damage causes the cell cycle arrest in G1, S and G2/M phase. Varying with this research, Yang Liu et al. (73) witnessed calycosin could induce cell cycle arrest in G0/G1 when treated for hepatocellular carcinoma (HCC) cells (73). It follows AKT signaling pathway. In our current research, S phase arrest is possible due to the inhibition of cell cycle progression which is due to the ant-proliferative effects of Cabozantinib (CNB) and control and target-based release profiling of CNB-PLGA-PSar-NPs. According to Shamim Akhtar Sufia et al. (74), curcumin and indole-curcumin analog-loaded polysorbate 80-stabilized PLGA nanoparticles have a great anticancer effect on SW480 colon cell lines (74). The size of the NPs that were made ranged from 50 to 150 nm. When nanoparticles were used to treat the SW480 cancer cell line, nuclear fragmentation, blocking of the cell cycle, stopping apoptosis, and metastatic biomarkers happened. The effects of Anti-EGFR conjugated Docetaxel loaded PLGA nanoparticles on the human lung carcinoma A549 cell line were studied by Jitendrakumar Patel et al. (75). They discovered that the cell cycle slowed down in the S phase with more cells in the G2/M phase and stopped in the G2/M phase. A549 cell lines, on the other hand, experienced a growth arrest in the G2/M phase when using the DTX-NPs system (75).

### 3.8 Flow cytometry

**Figure 6:**
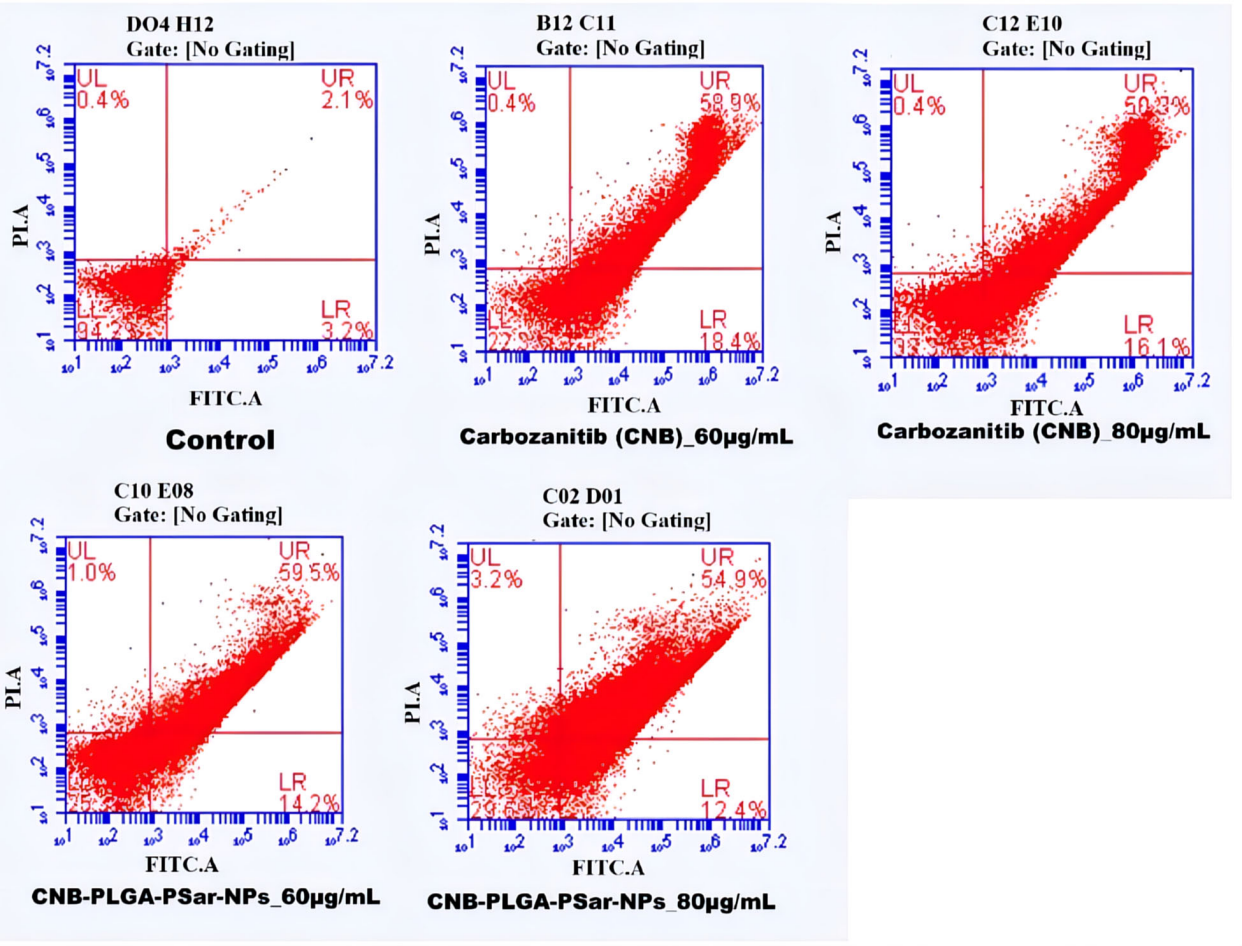
Flow cytometry analysis of HepG2 cells treated with different concentrations of CNB loaded CNB-PLGA-PSar-NPs and free CNB for 48 h, followed by staining with Annexin V-FITC and propidium iodide (PI). Results are representative of three independent experiments.

Flow cytometry was used to do an apoptosis assay to find out how significant the effects of the cell responses were (76). This study explains how cells respond to the given treatment. A paramount percentage of apoptosis was observed in Cabozantinib (CNB), and CNB-PLGA-PSar-NPs loaded group as compared to the untreated control group. The apoptosis percentage was found to be high for CNB-PLGA-PSar-NPs loaded group as compared to the free drug. Almost 20% apoptosis was recorded in the early stage, and 25% apoptosis was recorded in the late stage. Nearly 35% apoptosis was recorded in the late apoptosis chamber. Therefore CNB-PLGA-PSar-NPs possessed a higher therapeutic index and optimum anticancer effects (**Figure 6**). As per Salih Abdul Mahdi et al. (77), 5-FU loaded biocompatible polymeric magnetic nanoparticles and FA-DEX-SPION NPs exert synergistic effects for targeting intracellular delivery of 5-FU, apoptosis induction, and gene expression stimulation (77)

### 3.9 Real-time PCR analysis

**Figure 7:**
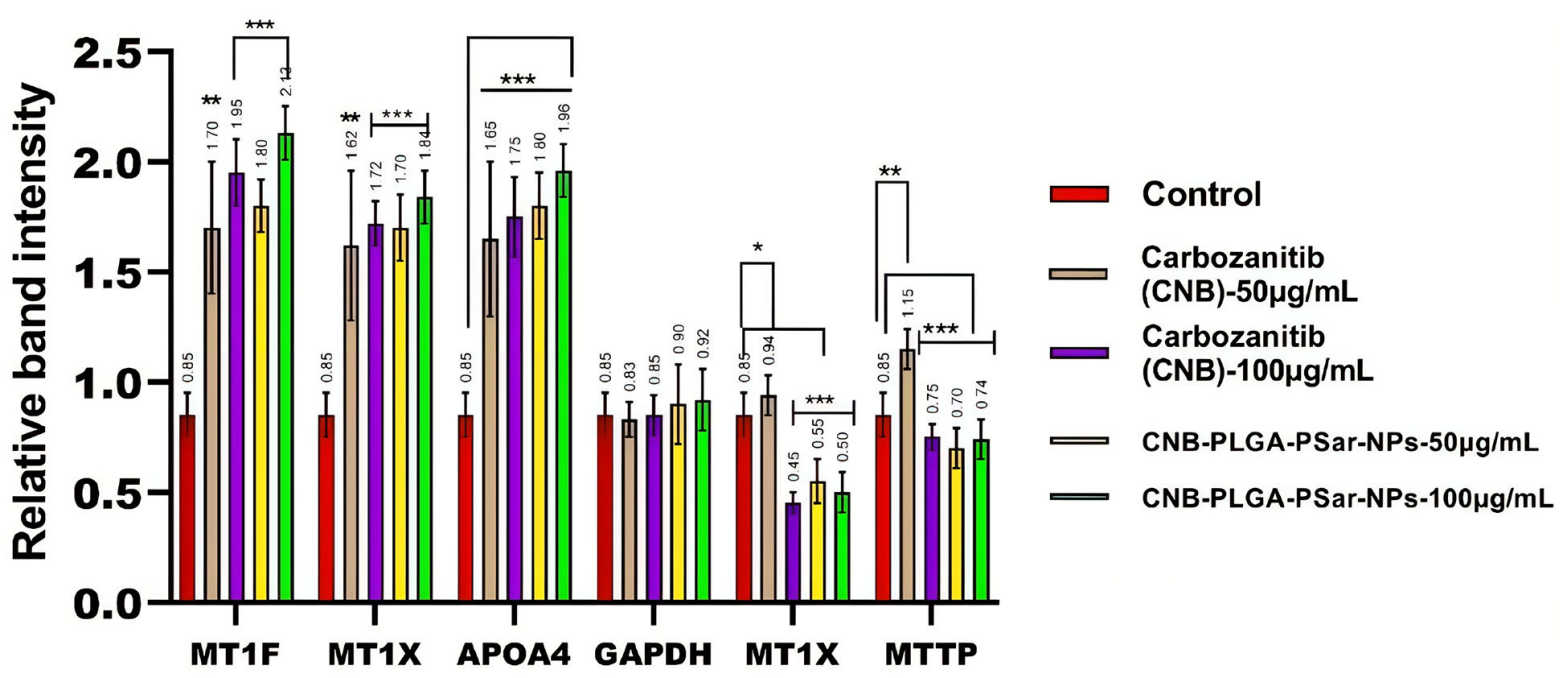
The expression levels of MT1F, MT1X, APOA4, MT1X, MTTP genes in relative to the reference gene; Glyceraldehyde 3-phosphate dehydrogenase (GAPDH) in HepG2 cancer cell line treated with the CNB-loaded CNB-PLGA-PSar-NPs and free CNB. *P< 0.05, **P< 0.01 and ***P< 0.001 vs. control was considered significant.

In this study, we investigated the effects of different concentrations of free Cabozantinib (CNB), CNB-PLGA-PSar-NPs, or a combination of the two on the genes in the human hepatoma HepG2 cell line (78). Affymetrix Human Genome 1.0 S. T arrays were utilized in the study to conduct a microarray analysis. The microarray results were validated using real-time RT-PCR and semi-quantitative RT-PCR. In HepG2 cells exposed for 24 hours to free Cabozantinib (CNB), CNB-PLGA-PSar-NPs at concentrations of 0, 25, and 55 µg/mL. The real-time PCR was used to examine the mRNA levels of MT1F, MT1X, GSTA1, and APOA4. According to the current findings, CNB-PLGA-PSar-NPs change the mRNA expression of the genes listed above much more than free CNB. In other words, when free Cabozantinib (CNB), CNB-PLGA-PSar-NPs-treated cells were compared to control cells, the expression of MT1F, MT1X, and APOA4 was significantly upregulated while the levels of MT1X and MTTP were decreased. The well-known metallothionein MT1F is essential for the stability of the genome, the cell cycle, and apoptosis. It has many different targets. In the presence of glutathione S-transferases, an apoptogenic factor, GST02 as an initiator and GSTA2 as an effector, it has been shown that most polymeric NPs promote penetrability transition, which in turn promotes apoptosis. In a previous study, the main proteins that control mitochondrial-mediated apoptosis were found to be from the MT1X family. The proapoptotic and antiapoptotic activities of MT1X and GSTA1 have been found. Cysteine proteases were successfully conserved by the family of cytoplasmic proteases known as caspases, which is essential in the apoptotic signaling pathway. The most important member of the family, MT1X, is essential for the induction of apoptosis. Microsomal triglyceride transfer protein (MTTP) overexpression in the liver is required for the hepatic secretion of lipoproteins containing apoprotein B (apoB). Inhibition of MTTP results in a decrease in plasma levels of apoB and hepatic triglyceride secretion (**Figure 7**). Overexpression, which is present in more than 45% of human hepatocellular carcinoma cells, causes hepatic cancer in transgenic mice. In this study, we demonstrated that upregulation of the tumour suppressor genes MT1F, MT1X, and APOA4 and downregulation of the genes MT1X and MTTP are the mechanisms by which free Cabozantinib (CNB) and CNB-PLGA-PSar-NPs kill HepG2 cells. However, it is essential to assess the novel therapeutic effects of prepared NPs based on CNB. Dorota Katarzyska-Banasik et al. (79) carried out a similar experiment and chose eight genes for validation: HPRT, HMBS, VIM, SDHA, TBP, RPL13, GAPDH, and 18S rRNA. The ideal combination of reference genes, according to geNorm, is TPP and SDHA (79). P53, P21, Caspase-9, and AKT-1 expressions were upregulated in FA-VNC-DEX-SPION Tera-1-treated cells, during RT-PCR studies, according to Sharafaldin Al-Musawi et al. (80). As per Shuang Liu et al. (81) findings, C-terminal binding protein 1 (CTBP1) was the most stable reference gene among 6 candidates evaluated by genorm and normalfinder in RT-qPCR analysis of tumor tissues from male hepatocellular carcinoma patients with hepatitis B infection and cirrhosis. The stability values for CTBP1 were 0.08 and 0.044 for genorm and normfinder, respectively. The authors recommend using CTBP1 as the best candidate reference gene for RT-qPCR in studies of HCC related to HBV infection.In our current research also MT1X and MTTP were downlreguated.Alltogather the study investigated the effects of different concentrations of free Cabozantinib (CNB) and CNB-PLGA-PSar-NPs on genes in the human hepatoma HepG2 cell line. The study used microarray analysis, real-time RT-PCR and semi-quantitative RT-PCR. The results showed that CNB-PLGA-PSar-NPs change the mRNA expression of certain genes more than free CNB. These changes led to the promotion of apoptosis, and the inhibition of MTTP which results in a decrease in plasma levels of Apolipoprotein B and hepatic triglyceride secretion. The study suggests that the upregulation of tumour suppressor genes and downregulation of certain genes are the mechanisms by which free Cabozantinib (CNB) and CNB-PLGA-PSar-NPs kill HepG2 cells.

### 3.10 Erythrocyte membrane integrity

**Figure 8:**
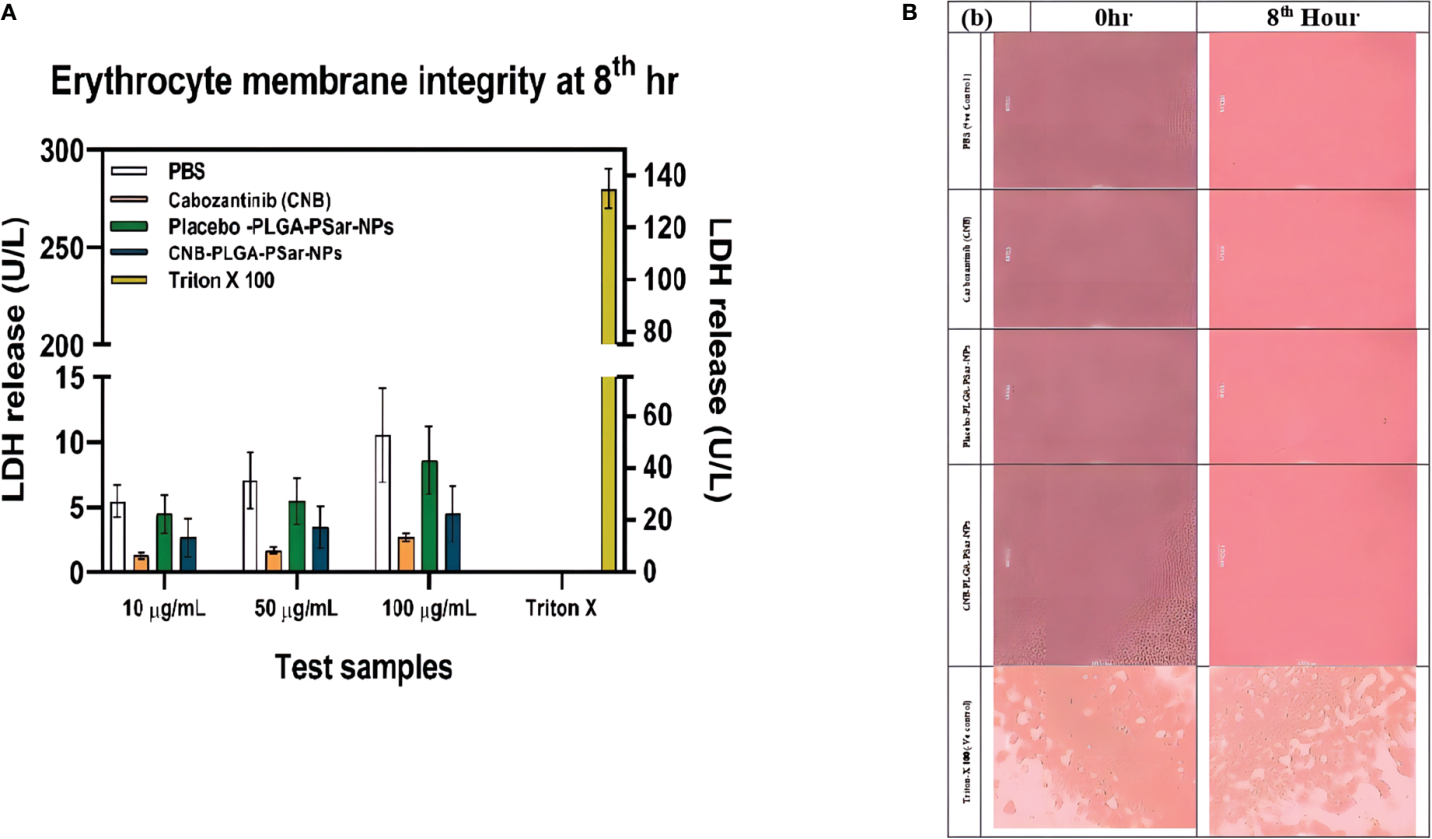
**(A)**. Amount of LDH release after treating with different concentration (10 µg/mL,50 µg/mL,100µg/mL) of Cabozantinib (CNB), PLGA-PSar-encapsulated Cabozantinib nanoparticles (CNB-PLGA-PSar-NPs), and placebo (PLGA-PSar-NPs) nanoparticles against PBS (posative control) Triton X 100(negative control) at 8th hour. **(B)**. Microsopic images of human erytocytic membrane after incorporation of CNB, PLGA-PSar-NPs, CNB-PLGA-PSar-NPs in the presence of posative and negative control.

In this investigation, centrifugation was employed to separate plasma and white blood cells from blood, leaving only erythrocytes floating in normal saline solution. Researchers were able to determine membrane integrity by measuring LDH enzyme concentrations. A defect in the erythrocyte membrane integrity produces an increase in LDH release. The amount of LDH released after treating 1 mL of erythrocyte suspension with phosphate buffer solution, Cabozantinib (CNB), PLGA-PSar-encapsulated Cabozantinib nanoparticles (CNB-PLGA-PSar-NPs) (Equivalent to 10, 50, and 100 µg/mL of Cabozantinib), and placebo (PLGA-PSar-NPs) nanoparticles equal to the volume of molecular weights of PLGA nanoparticles of Cabozantinib (CNB) were mentioned respectively. Surprisingly, after 8 hours of incubation, placebo formulations did not demonstrate a significant rise in LDH compared to phosphate buffer-treated samples. At these concentrations of test samples, erythrocytes are not harmed, according to the optional findings. Triton X 100, on the other hand, exhibits exceptionally significant LDH release, suggesting entire erythrocyte destruction. As a consequence, Cabozantinib (CNB) would not alter the integrity of erythrocyte membranes; thus, produced nanoparticles containing Cabozantinib (CNB) would be safe for intravenous administration from the perspective of erythrocyte safety (**Figures 8A, B**).

### 3.11 *In-vivo* anti-tumour efficacy study

**Figure 9:**
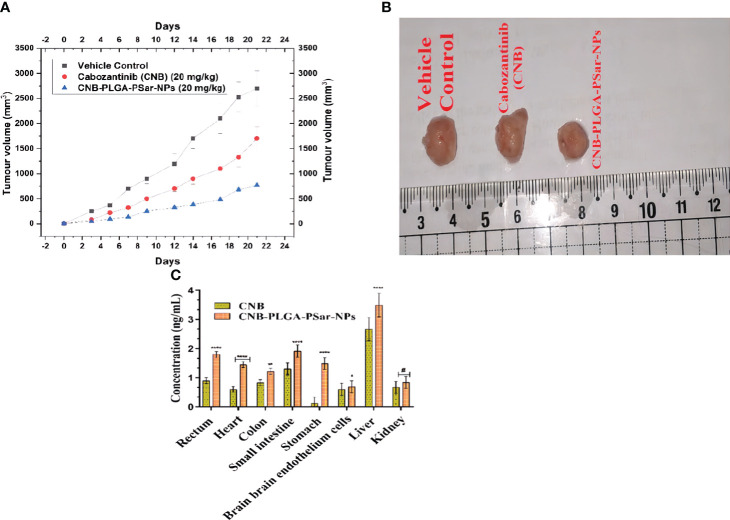
**(A)** Tumor regression study in HePG2 cells Xenograft model showing changes in relative tumour volume with time (days) of different groups of SCID (severe combined immunodeficiency disorder) mice (n=6) treated with Cabozantinib (CNB), CNB-PLGA-PSar-NPs. equivalent concentration 20 mg/kg, I.V.) **(B)** Isolation of tumours after 21 days of studies **(C) **Comparative *in vivo* biodistribution of Cabozantinib (CNB), CNB-PLGA-PSar-NPs in rectum, heart, colon, small intestine, stomach, brain endothelium, liver, and kidney after i.v. administration of 20 mg/kg. i.v. bolus dose; values are represented as mean± SD (n=6) (****) highly significance; p<0.00001, (***) moderately significance<0.0001, (**)partial significance; p<0.001, (*) less significance< 0.05, (#) statistically non-significance difference from CNB (p>0.05; all the formulations were compared against controlled group(CNB).

For the anticancer study, mice with severe combined immunodeficiency disorder (SCID) were given lateral tail vein injections of Cabozantinib (CNB) (20 mg/kg), CNB-PLGA-PSar-NPs (20 mg/kg). In animals with established HePG2 tumours, Cabozantinib (CNB) 20 mg/kg. i.v. which is a standard-of-care chemotherapy agent for liver cancer, decreased the size of the tumours by a large amount compared to the normal vehicle control. Compared to the vehicle control group, treatment with CNB reduced the size of tumours by 60.3% at 21 days (**Figure 9A**). Compared to the vehicle control, the lyophilized dose of CNB-PLGA-PSar-NPs (20 mg/kg. i.v.) showed an 85.67% reduction in the size of the tumour (**Figure 9B**). So, we can say that CNB-PLGA-PSar-NPs works better against tumours in biological systems. Surprisingly, when CNB-PLGA-PSar-NPs was injected into the animals, they lost weight and had great anti-tumour effects for a long time. This is a strong sign that the drug is targeting tumour tissues. So, polymeric nanoparticle systems of CNB made with PLGA and PSar have the benefit of reducing the higher dose-dependent toxicity of anticancer drugs while at the same time making the drugs work better against cancer. Shu Li et al. (2019 injected SKOV-3 cells into nude mice to induce ovarian cancer while developing folate-modified PLGA-based nanoparticles for the treatment of ovarian cancer. Compare to other groups, higher sensitivity against ovarian cancer is demonstrated by FA-PEG2000-PLGADTX/GEM NPs. Anti-Erb-BTNB-PCL-NPs demonstrated maximum *in vivo* anti-tumor efficacy in a similar pattern during our experiment.

### 3.12 Tissue distribution studies

The tissue distribution was studied by separating 18 SCID mice into three groups (each group had six mice). The drug and its Nano formulations were administered intravenously (i.v.) at a bolus dose of 20 mg/kg. Groups of mice were given a dosage and then killed eight hours later. The organs such as the liver, heart, colon, small intestine, stomach, brain, rectum muscle, and kidneys were harvested for study. Intact brain endothelial cells were isolated using a stereotaxic device (New Standard TM Stereotaxic Instrument (Model No.: 51500), USA). Tissue samples were washed in 0.9% sodium chloride solution, patted dry, then homogenised in cold physiological solution and filtered. The samples were analysed using RP-UHPLC (Dionex Ultimate, 3000). Bonferroni’s multiple comparisons test showed that CNB had a very significant distribution in liver, heart, stomach, and rectum tissues (p<0.05), suggesting that the nano-formulation (CNB-PLGA-PSar-NPs) would have a wider blood distribution and an appropriately high stomach resistance. Moreover, nanoparticles were found in a much more widespread fashion in the small intestine (p<0.05), indicating sufficient resistance in a basic environment. This also led to a significantly increased concentration of Nano formulations in the rectum. Nano-formulations don’t accumulate in the kidney, as shown by a p>0.05, lending credence to the hypothesis of rapid elimination. There is a significant decrease in drug accumulation in the brain, which is indicative of decreased drug absorption by Nanoformulations across the Blood-Brain Barrier (BBB) and, thus, decreased neurotoxicity. Bio-distribution results showed that CNB and CNB-PLGA-PSar-NPs were more widely distributed in the parenchymal hepatocyte tissue of mice. Which suggests that the Nanoformulations (CNB-PLGA-PSar-NPs) might be a useful tool for delivering CNB to the liver, which could be a promising approach to treating liver cancer (**Figure 9C**).

### 3.13 Pharmacokinetic study

**Figure 10:**
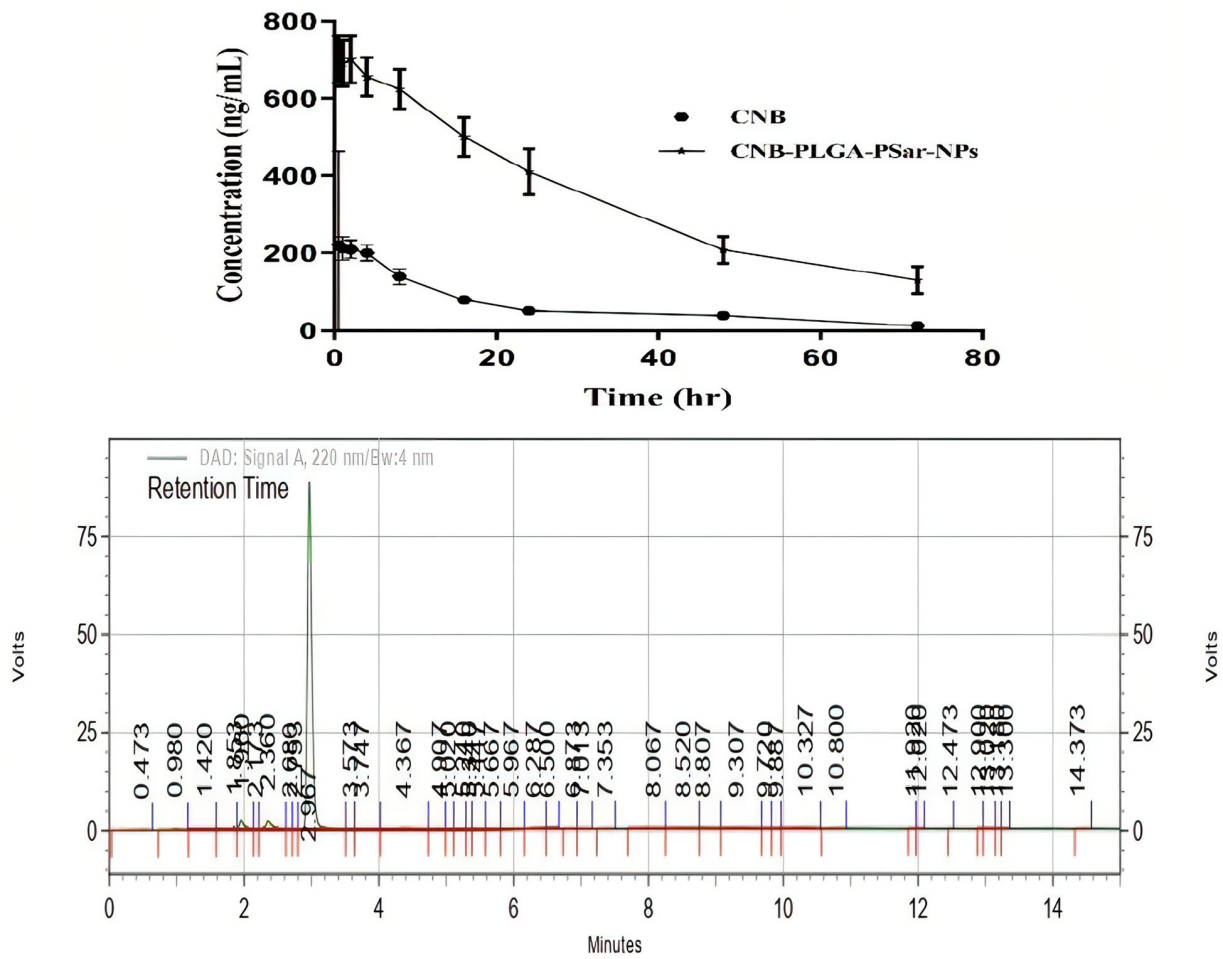
Concentration-time profiles in plasma of Cabozantinib (CNB) after i.v. bolus administration of CNB suspension and CNB loaded polymeric nanoparticles (CNB-PLGA-PSar-NPs) at 20 mg/kg to 6 male albino Wister rats (200–250 g) (n=3). The reverse phase HPLC method was used to figure out how much CNB was in the plasma. Put in an HPLC chromatogram showing the amount of CNB in the plasma. The CNB concentration in plasma was determined using reverse phase HPLC. Incorporate the plasma CNB concentration as an HPLC chromatogram.

Cabozantinib (CNB), a BCS class-II drug, has limited anticancer activity due to its low water solubility and poor bioavailability, despite its anti-proliferative effects against solid tumours. In order to increase the systemic bioavailability of Cabozantinib (CNB) and stop the spread of liver cancer, we developed CNB-PLGA-PSar-NPs, which are polymeric nanoparticles encapsulating Cabozantinib (CNB). The relevant pharmacokinetic parameters C_0_, C_max_, V_d_, t_1/2_, CL, AUC, and MRT are summarised in **Table 1**. Due to increased tissue bindings of Cabozantinib (CNB) encapsulated polymeric nanoparticles after intravenous bolus administration, as indicated by the pharmacokinetic profile of the drug and drug-encapsulated polymeric nanoparticles, the clearance (CL) value gradually decreased. Indicating that CNB-PLGA-PSar-NPs inhibited opsonin protein adhesion, the mean residence time (MRT) of CNB-PLGA-PSar-NPs was 50.0867 ± 3.78h, which was significantly longer than the MRT of Cabozantinib (CNB). This might be because nanoparticles are sneaky, allowing them to bypass the reticuloendothelial system and prolong systemic circulation. The statistically significant differences between free Cabozantinib (CNB) and CNB contained polymeric nanoparticles (CNB-PLGA-PSar-NPs) were compared using Dunnett’s student t-test. CNB-PLGA-PSar-NPs had significantly higher maximum plasma drug concentrations (C _max_), total clearance (CL), and area under the curve (AUC) than free CNB (p<0.001). Based on Figure, it was found that the plasma concentration of CNB-PLGA-PSar-NPs, which are polymeric nanoparticles coated with polylactic-co-glycolic acid (PLGA) and polysarcosine (PSar), was significantly higher than that of free CNB. Additionally, the surface finish of nanoparticles influences how well they circulate. The corona coating of PLGA and PSar over nanoparticles prevents plasma protein adsorption, prolongs the time that blood circulates through the body. Therefore, CNB-PLGA-PSar-NPs and all CNB-encapsulated polymeric nanoparticles could function as a potential Cabozantinib (CNB) delivery system to liver cancer (**Figure 10**).

**Table 1:** Pharmacokinetic parameters Cabozantinib (CNB), CNB-PLGA-PSar-NPs after intravenous administration of 20 mg/kg dose.

Parameters	CNB	CNB-PLGA-PSar-NPs
**C_0_(ng/mL)**	215.148 ± 16.32	713.032 ± 23.37
**C _max_ (ng/mL)**	241.313 ± 34.21	668.25 ± 56.62*
**V_d_ (ml)**	1522.30 ± 1.54	22342 ± 40.420
**t_1/2_(hr^-1^)**	05.21 ± 0.18	37.433 ± 2.25
**CL (mL/hr)**	124.51 ± 1.13	32.536 ± 8.49*
AUC (ng_*_mL/hr)	1956.73 ± 111.39	29926.234 ± 653.89*
**MRT (h)**	7.445 ± 0.24	43.0661 ± 10.58

Data are presented as mean ± SD (n=3) (*) statistically significantly different from ECF.

### 3.14 *In vivo* immunohistochemical study

**Figure 11:**
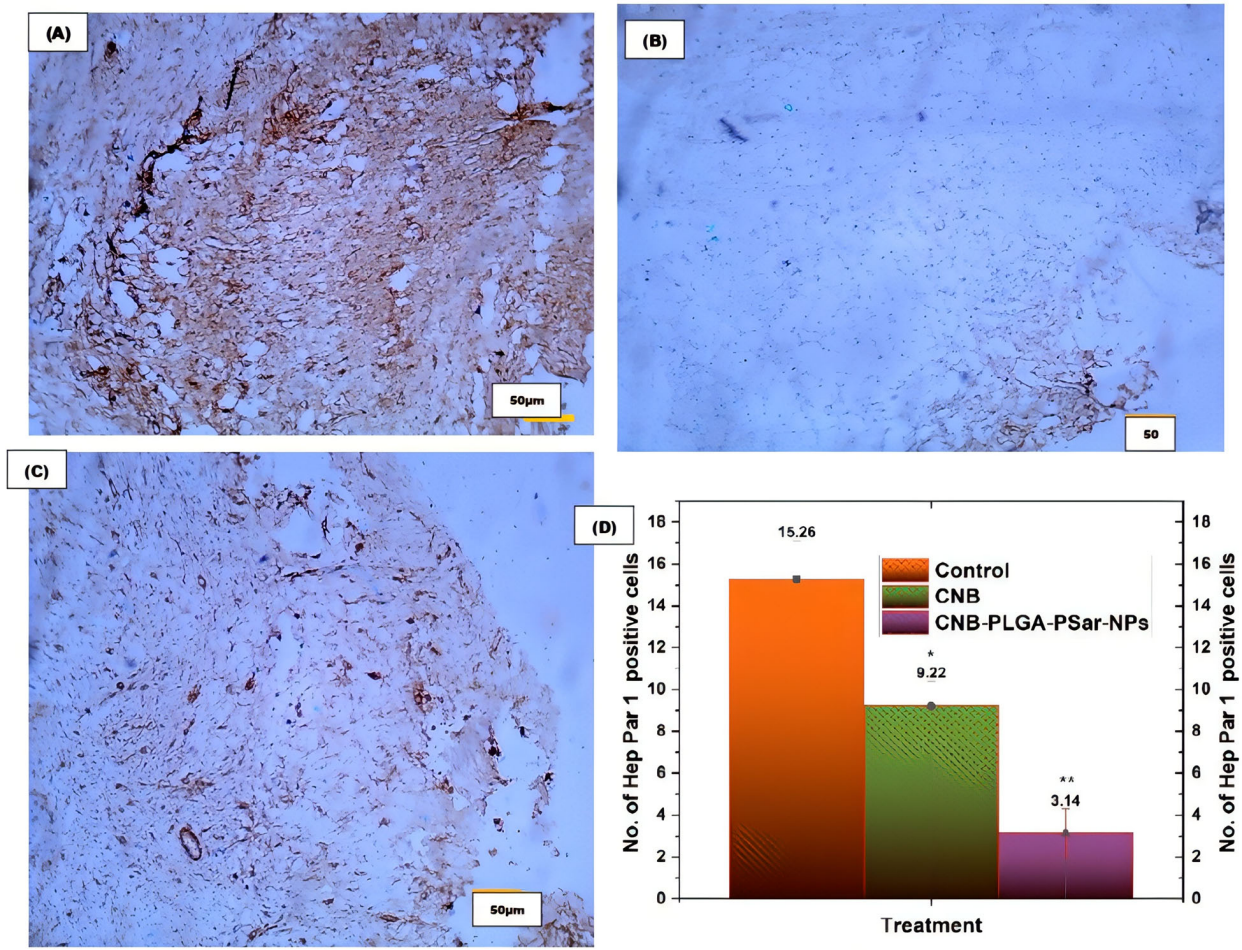
Immunohistochemical study for the Hep Par 1 expression by **(A)** control (PBS buffer treated), **(B)** free CNB, and **(C)** CNB-PLGA-PSar-NPs treated tumours. **(D)** The quantitative analysis of IHC results. Statistical comparisons were made using t-test; * partial significance; p < 0.001; ** moderately significance < 0.000. The treatment was compared with control (PBS buffer treated).

Tumour sections were immunohistochemically (IHC) stained for Hep Par 1 expression. This was done so that the proliferative ability of cancer cells could be evaluated *in vivo*. Hep Par 1 is a cell marker that can be found during all of the active phases of the cell cycle, including G1, S, G2, and M. It is widely used for the purpose of determining the rate of cell proliferation. A suboptimal response to chemotherapy is associated with high levels of expression of Hep Par 1, which is also linked to aggressive tumour behaviour, vascular invasion, and metastasis of tumours. IHC investigations for Hep Par 1 using paraffin-embedded xenograft cancer tissue blocks from mice with (A) control (PBS-treated) group, (B) CNB -treated group, and (C) CNB-PLGA-PSar-NPs -treated group are depicted in Figure. For the purpose of evaluating the IHC data, five random 400 µm × 400 µm square zones were placed on each sectioned slide. As can be seen in Figure, tumours that were treated with CNB-PLGA-PSar-NPs had a significantly lower Hep Par 1 labelling index (3.14 ± 1.71) than those that were treated with free CNB (9.22 ± 1.67) or the control group (15.26 ± 1.88), indicating that CNB-PLGA-PSar-NPs are effective in inhibiting the growth of tumours. This is supported by the fact that the control group had a significantly higher index. Based on these findings, it appears that CNB-PLGA-PSar-NPs play a significant part in the process of increasing the inhibition of tumour cell growth, causing the death of tumour cells, and demonstrating the ability to assist in the suppression of tumours (**Figures 11A–D**).

## 4 Discussion

The fabrication of nanoparticles composed of Poly D, L-lactic-co-glycolic acid (PLGA) and Polysarcosine (PSar) over Cabozantinib (CNB) has been studied for its potential in inducing apoptosis and cytotoxicity in human HepG2 hepatocellular carcinoma cell lines and in SCID female mice model. CNB is a tyrosine kinase inhibitor that is known to have anti-tumor activity, and the addition of PLGA and PSar to the CNB nanoparticles may enhance this activity.In the study, the CNB nanoparticles were fabricated using a double emulsion solvent evaporation method. The PLGA and PSar were then added to the nanoparticles as coating materials. The resulting nanoparticles were characterized for their size, shape, and surface charge. The nanoparticles were further evaluated for cytotoxicity and apoptosis-inducing ability of the CNB-PLGA-PSar-NPs nanoparticles in human HepG2 hepatocellular carcinoma cells. The results showed that the CNB-PLGA-PSar-NPs nanoparticles were more effective in inducing apoptosis and cytotoxicity in the cancer cells than CNB alone. Additionally, the study also found that the CNB-PLGA-PSar-NPs nanoparticles caused a significant downregulation of the expression of genes related to cell proliferation, migration and invasion. Polysarcosine (PSar) and PLGA nanoparticles are advantageous as a delivery system for cabozantinib (CNB) due to their ability to protect the drug from degradation and improve its solubility. Additionally, these nanoparticles can target specific cells or tissues, leading to increased drug efficacy and reduced toxicity. Furthermore, polysarcosine (PSar) and PLGA nanoparticles have been shown to have improved stability, which increases the shelf life of the drug and reduces the need for frequent dosing. The study of Poly D, L-lactic-co-glycolic acid, and Polysarcosine over Cabozantinib nanoparticles (CNB-PLGA-PSar-NPs) has been further confirmed for its excellent bio-compatibility and liver disposition using various techniques such as lactate dehydrogenase (LDH) assay, pharmacokinetics, tissue distribution, immune-histochemistry and *in-vivo* anticancer effects in SCID female mice. The LDH assay was used to evaluate the cytotoxicity of CNB-PLGA-PSar-NPs on normal human blood erythrocytes and the results showed that the CNB-PLGA-PSar-NPs treated erythrocytes generate less Lactate dehydrogenase in compared to CNB treated erythrocytes. Pharmacokinetic studies were conducted to evaluate the pharmacokinetics and tissue distribution of CNB-PLGA-PSar-NPs in male albino wister rats. The results showed that CNB-PLGA-PSar-NPs had a prolonged circulation time and were preferentially distributed to the liver, which is consistent with the excellent liver disposition of the nanoparticles. Immune-histochemistry was performed to evaluate the anticancer effects of CNB-PLGA-PSar-NPs on HepG2 hepatocellular carcinoma cells; signifying, CNB-PLGA-PSar-NPs play a noteworthy part in the process of increasing the inhibition of tumour cell growth, causing the death of tumour cells. The *in-vivo* anti cancer results showed that CNB-PLGA-PSar-NPs pointedly reduced the tumor volume and induced cancer cell death. Overall, these results confirm the excellent bio-compatibility, liver disposition and anticancer effects of CNB-PLGA-PSar-NPs in SCID female mice. These findings suggest that CNB-PLGA-PSar-NPs may be a promising therapeutic strategy for the treatment of hepatocellular carcinoma. Further studies are needed to fully understand the mechanisms of action of these nanoparticles and to optimize their design for *in vivo* use.

## 5 Conclusion

We created a nanocarrier system based on CNB-PLGA-PSar-NPs that were loaded with Cabozantinib (CNB), and we assessed it using SEM, TEM, AFM, FTIR, *in-vitro* drug release, and HepG2 cancer cells to determine its anticancer effect. We made nanosized, spherical CNB-PLGA-PSar-NPs particles with a small variety of sizes. The CNB-loaded CNB-PLGA-PSar-NPs had a robust cytotoxic effect on the HepG2 cancer cells. The fact that CNB-loaded CNB-PLGA-PSar-NPs have a more significant HepG2 cancer cell-killing effect than free CNB may be attributed to their sustained and prolonged drug release characteristics, high cellular internalization, and tumour targeting efficacy. Furthermore, it has been shown that the HepG2 cell lines were considerably affected by the CNB nanoformulation’s strong induction of apoptosis. Finally, it has been shown that CNB-loaded CNB-PLGA-PSar-NPs can trigger cell death since cancer cells treated with them have increased tumour suppressor and apoptotic gene activity compared to the free CNB group. The study prepared and tested CNB-PLGA-PSar-NPs nanoparticles for the first time for biocompatibility, biodistribution, and anti-tumor activity against the human HepG2 liver cancer cell line and SCID female mice. We performed lactate dehydrogenase assay, pharmacokinetic studies, tissue distribution studies, *in vivo* cancer activity in SCID female mice, and immunohistochemistry to confirm the biocompatibility, biodistribution, and anti-tumor activity of the prepared CNB-PLGA-PSar-NPs nanoparticles.Thus, our results demonstrate the potential of innovative CNB-loaded CNB-PLGA-PSar-NPs as a novel strategy for treating hepatocellular carcinoma and the potential of CNB-PLGA-PSar-NPs as a promising carrier in a CNB delivery system.

## Data availability statement

The raw data supporting the conclusions of this article will be made available by the authors, without undue reservation.

## Author contributions

Data curation, SB and VP. Funding acquisition, BP. Investigation, VP and BP. Methodology, SB. Resources, BP. Supervision, BP. Validation, VP. Writing – original draft, SB. Writing – review and editing, BP. All the authors have reviewed and approved the final manuscript. SB communicated to the journal.
